# Sequential polarization of macrophages: an immunomodulatory strategy for novel bone repair materials

**DOI:** 10.1093/rb/rbag144

**Published:** 2026-07-07

**Authors:** Xueqin Gao, Shuao Dong, Xiyue Zheng, Yunhui Si, Chao Zhang

**Affiliations:** School of Biomedical Engineering, Shenzhen Campus of Sun Yat-sen University, Shenzhen 518107, China; School of Biomedical Engineering, Shenzhen Campus of Sun Yat-sen University, Shenzhen 518107, China; School of Biomedical Engineering, Shenzhen Campus of Sun Yat-sen University, Shenzhen 518107, China; School of Biomedical Engineering, Shenzhen Campus of Sun Yat-sen University, Shenzhen 518107, China; School of Biomedical Engineering, Shenzhen Campus of Sun Yat-sen University, Shenzhen 518107, China

**Keywords:** osteoimmunomodulation, macrophage, sequential polarization, bone repair materials

## Abstract

Within the biomaterial-constructed bone repair microenvironment, intricate crosstalk among immune, osteogenic and vascular cells is essential for maintaining local osteoimmune homeostasis and driving tissue regeneration. Macrophages act as the central orchestrators within this osteoimmune microenvironment. As the first responders to injury, they exert decisive spatiotemporal control over the initiation, maintenance and resolution of inflammation, while bridging osteogenesis, angiogenesis and matrix remodeling. Critically, their dynamic phenotypic transition, specifically the sequential polarization from M1 to M2 phenotypes, directly mediates immune microenvironment remodeling. This sequential shift bidirectionally regulates osteoprogenitor differentiation and endothelial cell function via the secretion of specific cytokines and extracellular vesicles, thereby achieving a dynamic equilibrium between the immune response and tissue regeneration. Consequently, precisely modulating the sequential polarization of macrophages has emerged as a targeted and highly promising strategy for the development of novel bone repair biomaterials. This review systematically summarizes the regulatory mechanisms of distinct macrophage phenotypes in bone regeneration and highlights recent translational advances in designing osteoimmunomodulatory biomaterials tailored for sequential polarization. By critically examining current challenges and future trajectories, this review aims to provide profound insights into the multidimensional macrophage activation spectrum and accelerate the development and optimization of next-generation immunomodulatory biomaterials for robust bone regeneration.

## Graphical abstract

**Graphical Abstract:**
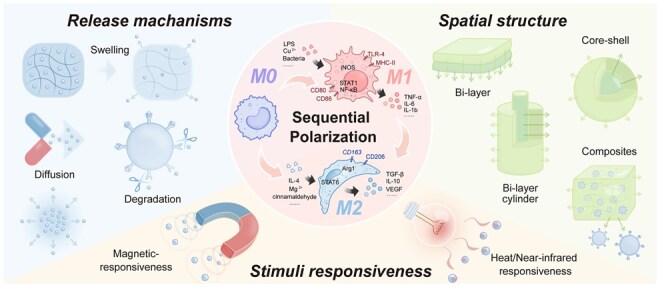

## Introduction

Although bone possesses a certain regenerative capacity, bone defects are difficult to heal spontaneously when the injury exceeds a critical size, and the healing process is further constrained by multiple factors. Consequently, the treatment of large bone defects arising from trauma, infection or cancer still represents a significant challenge worldwide. Currently, autogenous bone grafting remains the clinical gold standard, along with allografting and xenografting. However, the clinical adoption of these grafting methods is hindered by limited donor availability and concerns over disease transmission. Therefore, the design of bone biomaterial implants represents a promising approach for treating bone defects.

Traditional bone repair implants have mainly aimed to optimize material bioactivity and mechanical properties, mediating the adhesion, proliferation and osteogenic differentiation of osteoblast lineage cells, such as mesenchymal stem cells (MSCs), through physical/chemical cues, including stiffness, viscoelasticity, porosity and cytokines, thereby promoting new bone formation. However, the excessive emphasis on mechanical support and osteogenic activity, while neglecting the critical regulatory role of the host immune response, often leads to issues such as implant–bone interface fibrosis or poor osseointegration in the application of conventional bone repair materials [1]. To address this challenge, bone repair materials with ‘immunoactive modulation’ have emerged.

In 2016, Chen *et al*. [2] first systematically proposed the concept of ‘osteoimmunomodulation’ (OIM), indicating that implant materials function indirectly by regulating MSC osteogenic differentiation through macrophage-derived factors like bone morphogenetic protein-2 (BMP-2) and vascular endothelial growth factor (VEGF), rather than through the traditional paradigm of direct stimulation of osteoblasts by materials. This explicitly marked the transition of bone repair biomaterials from ‘immuno-inert’ to ‘immunoactive modulation’. Since then, the field of OIM has advanced rapidly: in terms of macrophage polarization regulation, pro-inflammatory macrophages dominate the early inflammatory response, whereas anti-inflammatory macrophages initiate and regulate subsequent tissue repair and matrix remodeling [3]. Regarding immune cell network coordination, Tsukasaki and Takayanagi [4] emphasized the key coordinating roles of T cells, B cells and innate immune cells-alongside macrophages-in bone homeostasis regulation. In terms of multidimensional material strategies, approaches such as the modulation of surface topography and chemical functionalization [5, 6], delivery of bioactive ions and molecules [7, 8] and the development of smart responsive materials [9] have been employed to regulate the immune microenvironment for bone repair, thereby promoting bone regeneration. Collectively, these efforts have progressively established a systematic research framework centered on macrophages as the core hub, integrating the immune-osteogenic-angiogenic cascade.

Macrophages, as the core regulatory cells of the osteoimmune microenvironment, play an irreplaceable role throughout the entire bone process. In the early stage of bone injury, macrophages rapidly infiltrate the defect site, obtain energy through enhanced glycolysis, efficiently clear pathogens and necrotic tissue and secrete pro-inflammatory cytokines such as tumor necrosis factor-α (TNF-α), interleukin-1β (IL-1β) and IL-6 to initiate the repair process. Meanwhile, they recruit MSCs and endothelial progenitor cells through signaling molecules including C-C Motif Chemokine Ligand 2 (CCL2) and VEGF, establishing the cellular foundation for subsequent repair. As for the tissue formation phase, macrophages transition toward a pro-regenerative phenotype, secreting anti-inflammatory factors such as IL-10 and transforming growth factor-β (TGF-β), as well as osteogenic factors (e.g. BMP-2, VEGF), thereby recruiting osteoprogenitor cells, inducing angiogenesis and promoting bone matrix formation [10–12]. Notably, macrophage phenotypic polarization is not a static binary switch, but rather a dynamic and orderly evolution across spatiotemporal dimensions. Specifically, it exhibits a sequential polarization pattern, shifting progressively from an initial M1-dominant state to an M2-dominant state. According to the existing literature, the optimal time window for this transition is 3–7 days post-injury: M1 polarization peaks within 1–3 days post-injury, primarily responsible for pathogen clearance and phagocytosis of tissue debris, thereby laying the necessary foundation for subsequent repair; M2 polarization gradually becomes predominant after 3–7 days, initiating tissue repair and bone regeneration [13–17]. This temporal transition is vital to successful bone repair: excessive prolongation of the M1 phase or failure to transition to the M2 phase can lead to chronic inflammation, fibrosis and nonunion; conversely, premature dominance of the M2 phase without an adequate M1 initiation phase may compromise pathogen clearance and the initial driving force for tissue remodeling, similarly impairing orderly repair [18]. On this basis, the ability to precisely induce sequential polarization of macrophages has emerged as a key criterion for evaluating the immunomodulatory performance of novel bone repair materials.

**Figure 1:**
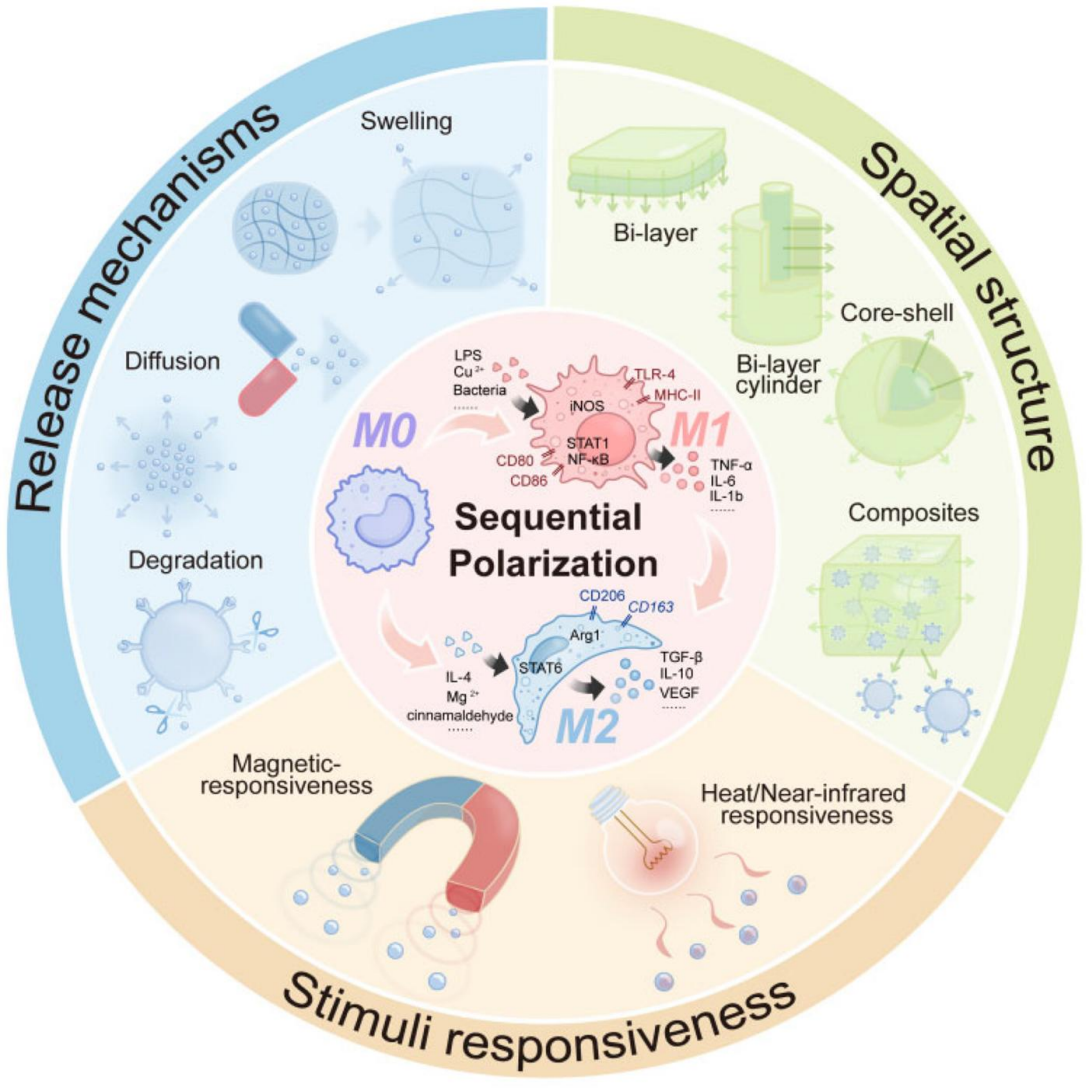
Strategies for regulating the sequential polarization of macrophages.

Herein, we introduce the two classical phenotypes of macrophages in OIM and their respective functions, emphasizing that both phenotypes are necessary for vascularization and bone regeneration. We also review existing strategies and research advances for regulating macrophage sequential polarization by osteoimmunomodulatory biomaterials (Figure 1), as well as the adaptations of these strategies under different pathological conditions. Finally, the challenges and future directions in this field are discussed. We hope this review will enhance understanding of the critical role of macrophages in bone repair within the context of OIM, highlight the potential of sequential macrophage polarization as a strategy that aligns with the natural bone repair process for osteoimmunomodulatory applications, and provide insights for developing future osteoimmunomodulatory biomaterials.

## The phenotype and function of macrophages

### Origins and classification of macrophages

As part of the mononuclear phagocyte system, macrophages are broadly distributed throughout the blood and tissues, serving critical functions in immune defense, surveillance and homeostasis. Macrophage polarization is a process through which macrophages acquire specific phenotypes and functionalities in response to microenvironmental stimuli and signals [19]. A thorough understanding of the origin, classification system and functional spectrum continuity of macrophages serves as the theoretical foundation for designing osteoimmunomodulatory materials and achieving sequential macrophage polarization.

Macrophages exhibit high heterogeneity, with their origins primarily encompassing embryo-derived tissue-resident macrophages and bone marrow-derived monocyte-derived macrophages. Tissue-resident macrophages colonize specific organs before birth and maintain their population through local self-proliferation in adulthood, primarily participating in tissue development, homeostasis maintenance and post-injury reparative responses [20]. Monocyte-derived macrophages migrate into tissues following blood circulation and differentiate into functionally diverse macrophages, exhibiting greater activity in infection and inflammation, with a predominantly pro-inflammatory response [21]. A specialized population of tissue-resident macrophages, termed osteomacs, resides in the bone lining cell layer of the periosteum and endosteum. These cells not only participate in physiological bone remodeling but also recruit monocyte-derived macrophages to bone injury sites in response to damage signals, playing a critical role in bone formation and bone homeostasis maintenance [21, 22]. Of note, although macrophages from different origins exhibit variations in their transcriptomic profiles and functions, they often display highly similar functional polarization and effector responses under identical microenvironmental stimuli. For example, both the RAW264.7 cell line and osteoclast precursor cells can effectively polarize into an M2-like phenotype with pro-osteogenic and anti-inflammatory functions in response to IL-4 stimulation [23]. This functional convergence is a core manifestation of macrophage plasticity and provides a feasibility basis for sequential polarization-based osteoimmunomodulatory strategies.

At the level of functional classification, the traditional theoretical model categorizes macrophages into two types based on differences in activation patterns and functions: M0 macrophages polarize to the M1 phenotype when stimulated by lipopolysaccharide (LPS) and Th1-type cytokines such as interferon-γ (IFN-γ) and TNF-α, whereas they differentiate into the M2 phenotype when stimulated by Th2-type cytokines such as IL-4. M1 macrophages, also known as classically activated macrophages, exhibit antibacterial and antitumor activities, express or secrete markers including inducible nitric oxide synthase (iNOS), TGF-α, IL-1β, IL-8 and IL-6, and exert pro-inflammatory and immunostimulatory effects. M2 macrophages, also referred to as alternatively activated macrophages, secrete a variety of cytokines such as IL-4, IL-10 and TGF-β, and play key roles in inflammation resolution, tissue repair and tumor progression [24]. However, this binary model has proven to be overly simplistic. Recent studies have clearly demonstrated that macrophages exhibit a continuous functional spectrum *in vivo* rather than discrete categories. Among these, the M2 phenotype has been further subdivided into at least five subtypes: M2a, driven by IL-4 or IL-13, is primarily involved in tissue repair and fibrotic processes; M2b is generated upon combined stimulation by immune complexes and Toll-like receptors, exhibiting both pro-inflammatory and anti-inflammatory characteristics; M2c is induced by IL-10 or TGF-β and displays potent immunosuppressive activity; M2d relies on adenosine signaling and mainly promotes angiogenesis and tumor progression; whereas M2f is induced by the phagocytosis of apoptotic cells and secretes various anti-inflammatory mediators to maintain vascular stability [17, 25–27]. Of note, the aforementioned subtypes often exhibit overlapping functions and molecular expression profiles, further highlighting the dynamic continuity and complexity of macrophage phenotypes. Therefore, while this review adopts the M1/M2 framework for simplified discussion, it also acknowledges the limitations of this model and will subsequently delve deeper into the multidimensional nature of the macrophage functional spectrum.

### Macrophages mediate immune regulation

Macrophages serve as essential components of the immunoregulatory system, exhibiting high heterogeneity and functional plasticity across different stages of the immune response [28]. Depending on the microenvironmental signals, macrophages can polarize into M1 and M2 phenotypes, which mediate pro-inflammatory and anti-inflammatory responses, respectively [29]. Furthermore, macrophages are capable of constructing immune cell niches through the secretion of chemokines, bridging adaptive immunity via antigen presentation and integrating immune signals through metabolic reprogramming to finely tune the intensity and progression of immune responses [30].

Regarding the initiation and resolution of inflammatory responses, macrophages can polarize into M1 and M2 phenotypes, which mediate pro-inflammatory and anti-inflammatory reactions, respectively. Upon stimulation with LPS and IFN-γ, macrophages polarize toward the M1 phenotype through the coordinated actions of the nuclear factor-κB (NF-κB) and signal transducer and activator of transcription 1 (STAT1) signaling pathways, and highly express pro-inflammatory cytokines such as TNF-α, IL-1β and IL-6, thereby activating various immune cells and driving Th1-type immune responses [31]. In contrast, upon induction by cytokines such as IL-4 and IL-13, macrophages polarize toward the M2 phenotype via STAT6 and peroxisome proliferator-activated receptor-γ (PPAR-γ) signaling, and secrete anti-inflammatory cytokines including IL-10 and TGF-β to promote tissue repair and inflammation resolution [32]. The macrophage phenotypes are not fixed but are in a dynamic equilibrium. Studies have shown that multiple intracellular signaling pathways regulate macrophage polarization states by integrating metabolic and inflammatory signals. For instance, ubiquitination modulates the transition between M1 and M2 phenotypes by regulating E3 ligases and deubiquitinases activity, thereby influencing the duration and intensity of immune responses [33].

In terms of immune cell recruitment and spatial organization, macrophages construct specific immune microenvironments through the secretion of chemokines [34]. In the tumor microenvironment (TME), macrophages exhibit high functional heterogeneity. Studies have shown that CCL2^+^ stromal macrophages promote tumor progression by recruiting pro-tumorigenic macrophages originating from monocytes. In contrast, C-X-C Motif Chemokine Ligand 13 (CXCL13^+^) and CXCL9^+^ tissue-resident macrophages participate in the formation of tertiary lymphoid structures and enhance anti-tumor immune responses by recruiting T cells and B cells [35]. This chemokine-defined macrophage niche mechanism reveals the key function of macrophages in spatially organizing immune cells.

Furthermore, as professional antigen-presenting cells, macrophages display co-stimulatory markers like CD80 and CD86, along with major histocompatibility complex class II (MHC-II) molecules. Through antigen phagocytosis and processing, macrophages present antigenic peptides to CD4^+^ T cells, thereby initiating adaptive immune responses [36]. In tumor-draining lymph nodes, monocyte-derived macrophages and dendritic cell subsets can exhibit immunosuppressive antigen-presenting functions, inducing T cell anergy or regulatory T cell differentiation, thereby dampening antitumor immunity [37].

### Macrophages mediate angiogenesis

Bone is richly vascularized; its development and repair occur through two distinct processes: intramembranous ossification (IMO) and endochondral ossification (ECO), with angiogenesis representing a common step that typically precedes bone formation. During IMO, MSCs aggregate and condense; a subset of these MSCs differentiates into capillaries, while the remaining cells differentiate into osteoblasts and form ossification centers. Abundant extracellular matrix is secreted, which gradually mineralizes and matures. Throughout this process, blood vessels provide sufficient oxygen and nutrients, extending from the ossification center toward the peripheral bone matrix to support osteocytes and newly formed bone tissue. The role of blood vessels is even more prominent during ECO. On one hand, avascular cartilage relies on blood vessels within the surrounding perichondrium for nutrient and gas exchange, and osteoblasts migrate from these perichondrial vessels to the cartilage margin to form the bone collar. On the other hand, following hypertrophy of chondrocytes within the cartilage template and their secretion of osteogenic and angiogenic factors, invasion of the vascular system delivers key cells, such as MSCs, osteoblasts and hematopoietic stem cells, to the primary ossification center. A similar process occurs in the epiphyses to form secondary ossification centers, ultimately culminating in bone formation. In summary, blood vessels serve as the structural template for the skeleton [38], mediating nutrient and gas exchange, cell migration and communication between bone tissue and its surrounding environment.

Macrophages serve as key mediators of blood vessel formation. Upon arriving at the injury site, macrophages phagocytose and clear debris and dead cells, and through the secretion of key factors such as VEGF, fibroblast growth factor (FGF) and platelet-derived growth factor (PDGF), they are deeply involved in the entire process of matrix remodeling, vascular sprouting and vessel maturation. For instance, M2 macrophages secrete TGF-β and PDGF-BB, which promote the migration of endothelial cells (ECs) and pericytes, respectively, enhance intercellular communication, and facilitate angiogenesis and vessel stabilization. In addition, M2 macrophages secrete matrix metalloproteinases (MMPs), which stimulate matrix degradation and angiogenesis, thereby accomplishing vascular remodeling. This process is indispensable for angiogenesis [39, 40]. Furthermore, crosstalk between M2 macrophages and ECs further enhances their pro-angiogenic functions [41].

However, the impact of M1 macrophages on the vascularization process remains a subject of debate. First, regarding the mechanisms by which M1 macrophages influence vascularization, numerous studies using conditioned medium from M1 macrophages in ECs tube formation assays have demonstrated that M1 macrophages can promote angiogenesis [12, 42]. In contrast, co-culture studies of macrophages with distinct phenotypes and ECs have yielded opposing results, showing that the M1 phenotype suppresses tube formation. This inhibitory effect has been attributed to direct cell–cell contact between macrophages and ECs [42]. Moreover, in other studies, sustained M1 polarization has been shown to inhibit angiogenesis [43]. These observations suggest that the pro-angiogenic or anti-angiogenic effects of M1 macrophages are influenced by multiple factors, including the duration of polarization, direct cell-cell contact and paracrine signaling.

In a recent study, Spiller *et al*. [17] employed Transwell co-culture assays and an *in vivo* three-dimensional (3D) vascularization model to demonstrate that M1, M2 and their subtypes (M2a, M2c and M2f) exert distinct roles at specific stages of the vascularization process. M1 macrophages secrete VEGF, initiating macrophage-associated angiogenic responses, promoting the expression of genes associated with EC sprouting, and facilitating EC migration and proliferation. Subsequently, the M2a, M2c and M2f subtypes mediate EC–pericyte interactions, regulate vascular sprouting and promote vessel maturation, respectively, participating in multiple processes including vessel stabilization and remodeling. Notably, this study also found that prolonged exposure to the M1 phenotype (3 days) led to the regression of *in vivo* angiogenesis. Furthermore, numerous studies have shown that excessive VEGF impairs pericyte recruitment and disrupts normal vascular morphology [44], whereas PDGF-BB prevents aberrant vascular growth induced by high doses of VEGF, mediates pericyte recruitment and promotes vessel maturation and stabilization [45]. Collectively, these findings indicate that sequential activation of the two phenotypes in combination promotes vascularization.

In summary, the repair and regeneration of bone tissue are highly dependent on angiogenesis. Macrophages, serving as the ‘dynamic commanders’ of the angiogenic process, orchestrate a comprehensive regulatory network for angiogenesis through their sequential activation: M1 macrophages exert complex dual regulatory effects, whereas M2 macrophages stably promote angiogenesis through synergistic actions involving multiple factors. Angiogenesis is a complex cascade process that requires the involvement of various cytokines at distinct time points [46]. Accordingly, the regulation of angiogenesis by macrophages is not a simple M1/M2 dichotomy, but rather a dynamic equilibrium shaped by the polarization state, duration of action and mode of cellular interactions.

### Macrophages mediate osteogenesis

The complex process of bone regeneration and repair is achieved through the coordinated regulation of numerous cell types within the osteoimmune microenvironment. Being central to innate immunity, macrophages play an irreplaceable part in the process of osteogenesis, governing MSC osteogenic differentiation, bone matrix mineralization and bone tissue regeneration through processes such as phenotypic polarization, paracrine signaling, intercellular crosstalk and the integration of mechano-biochemical signals. Similar to angiogenesis, the core basis of macrophage-mediated osteogenesis lies in the dynamic temporal polarization of M1/M2 phenotypes. During the initial phase of bone injury, pro-inflammatory M1 macrophages are rapidly recruited to clear necrotic tissue, initiate an appropriate inflammatory response and recruit MSCs and osteoprogenitors, thereby activating the regenerative cascade. Following inflammation resolution, macrophages polarize toward the reparative M2 phenotype, exerting anti-inflammatory, tissue-remodeling and osteogenic inductive functions, representing the primary effector subset that promotes bone formation.

Although M1 macrophages secrete a wide range of inflammatory cytokines and primarily exhibit pro-inflammatory characteristics, their functions extend beyond the mere promotion of inflammation. The cytokines they secrete, such as TNF-α, IL-1β and IL-6, can also promote the migration, proliferation and osteogenic differentiation of osteoprogenitors toward the injury site [47]. Growth factors secreted by M1 macrophages, including PDGF and FGF, recruit neutrophils, MSCs and fibroblasts to the injured area. Additionally, TGF-β can gradually attenuate inflammation by inhibiting the NF-κB pathway, promoting the transition of macrophages toward the reparative M2 phenotype and inducing arginase-1 (Arg-1) expression to counterbalance iNOS activity, thereby maintaining the balance between inflammation and repair [18, 48]. Furthermore, M1 macrophages secrete oncostatin M (OSM) via the COX-2/PGE2 pathway. As a key cytokine of the IL-6 family involved in macrophage-driven osteogenesis, OSM activates the transcription factor STAT3 by engaging type II receptors on MSCs, thereby inducing osteoblast differentiation programs and promoting osteogenesis. Concurrently, OSM enhances bone matrix synthesis and mineralization, contributing to the regulation of bone resorption and the maintenance of bone homeostasis [49, 50].

After M1 macrophages have launched the bone repair program during the initial phase, M2 macrophages begin to exert their functions at appropriate time points. At this stage, M2 macrophages exhibit dual roles [18, 48, 51–55]. On one hand, M2 macrophages release cytokines including BMP-2, insulin-like growth factor-1 (IGF-1), IL-10 and TGF-β, thereby activating Smad1/5/8 signaling and enhancing the expression of key osteogenic transcription factors such as Runt-related transcription factor 2 (Runx2) and Osterix. They also secrete VEGF with spatiotemporal specificity, directing ECs to build 3D tubular structures, which then function as pathways for delivering oxygen, nutrients and osteoprogenitors. On the other hand, M2 macrophages also exert precise regulation over osteoclast activity by tuning the RANKL/OPG ratio, which ensures that bone formation and resorption remain properly coupled [56]. Furthermore, M2 macrophages upregulate the expression of Sp7 transcription factor (Sp7), an essential regulator of MSC osteogenic commitment and enhance the expression of genes associated with matrix deposition and mineralization in MSCs [12].

Macrophages also regulate osteogenesis through other signaling networks. For instance, Macrophage Scavenger Receptor 1 (MSR1) enhances the osteogenic differentiation capacity of BMSCs by mediating the PI3K/AKT/GSK3β/β-catenin signaling pathway, and upregulates the expression of proliferator-activated receptor gamma coactivator 1-alpha (PGC1α) to promote mitochondrial biogenesis and oxidative phosphorylation, thereby facilitating M2 polarization [57]. Under mechanical stimulation, Csf1 synthesized via the Piezo mechanosensitive ion channel 1 (Piezo1) signaling pathway induces periosteal-resident CD68^+^F4/80^-^ macrophages to differentiate into CD68^+^F4/80^+^ macrophages, which in turn promote bone formation by expressing and secreting thrombospondin-1 (Thbs1) to activate TGF-β1 [58]. Macrophages from obese bone marrow secrete microRNA-containing extracellular vesicles (BMM-EVs) that lead to skeletal degeneration, whereas lean BMM-EVs can rejuvenate the skeleton in obese recipients. This finding reveals that macrophage-derived exosomes, such as those carrying miR-140 and miR-378a, can remotely regulate the osteogenic lineage commitment of stem cells [59].

In summary, macrophages coordinately regulate osteogenic differentiation through multiple pathways: they remodel the immune microenvironment via M1-mediated pro-inflammatory and M2-mediated anti-inflammatory responses, thereby preventing chronic inflammation from inhibiting osteogenesis; they facilitate the guided osteogenic commitment of MSCs while inhibiting adipogenic differentiation via paracrine and contact-mediated signals, thereby regulating stem cell fate; they secrete matrix metalloproteinases such as MMP-9 and MMP-13 to degrade damaged collagen and mineralized matrix, and secrete factors including TGF-β1 and BMP-2 to promote the deposition and mineralization of new matrix, thereby facilitating bone matrix remodeling; and they promote angiogenic–osteogenic coupling to provide nutritional support for bone mineralization. Notably, macrophage depletion attenuates the anabolic effects of parathyroid hormone (PTH) and severely impairs the progression of bone healing [6, 60].

### Macrophages mediate coupling between angiogenesis and osteogenesis

**Figure 2:**
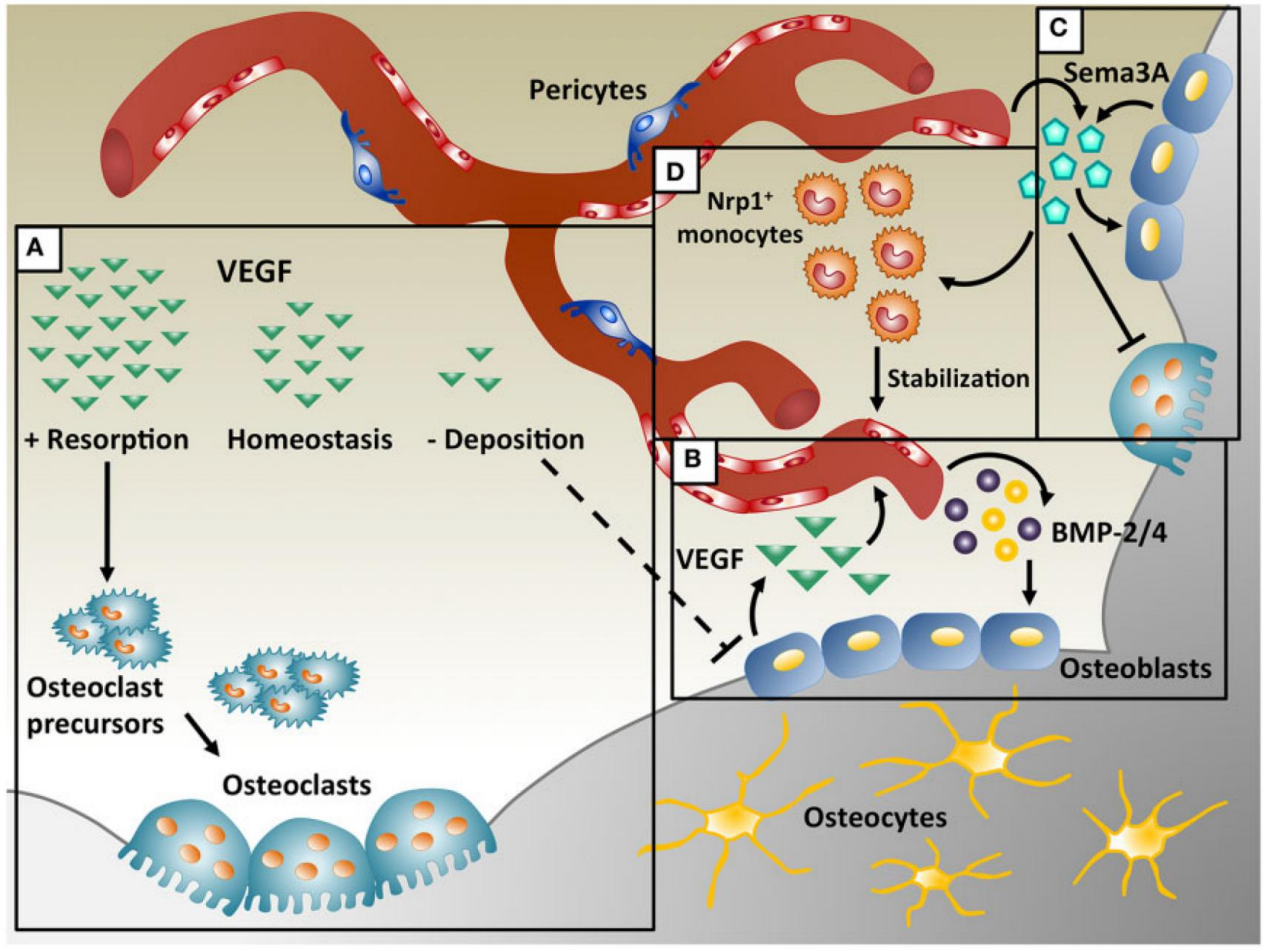
The coupling between angiogenesis and osteogenesis, with VEGF serving as a key mediating cytokine [38]. (**A**) Optimal VEGF levels maintain bone homeostasis. (**B**) Reciprocal crosstalk between osteoblasts and ECs involves VEGF and BMP-2/4 signaling. (**C**) VEGF upregulates Sema3A expression in ECs while inhibiting osteoclast differentiation. (**D**) Sema3A facilitates vascular stabilization by recruiting Nrp1^+^ monocytes.

Bone tissue repair is dependent on the coupling between angiogenesis and osteogenesis. Within this process, immune cells, macrophages in particular, play a modulatory role. Studies have shown that during bone development, there exists a bone-specific microvascular subtype termed type H vessels (CD31^+^EMCN^+^), whose distribution closely overlaps with regions of active osteogenesis, and which is recognized as a key vascular subtype participating in the coupling of angiogenesis and osteogenesis [61, 62] (Figure 2). M2 macrophages actively contribute to type H vessel formation through the release of pro-angiogenic factors, including VEGF and PDGF-BB. In turn, type H endothelial cells further reinforce the regenerative phenotype of macrophages through pathways such as hypoxia-inducible factor 1-alpha (HIF-1α)/VEGF and Notch, while directly stimulating the proliferation and differentiation of osteoprogenitors, thereby forming an ‘immune-vascular-osteogenic’ synergistic network [63–65]. Kohara *et al*. [66] depleted macrophages in a bone defect mouse model using clodronate liposomes, and observed impaired formation of type H vessels and disrupted angiogenesis-osteogenesis coupling, demonstrating that macrophages are essential for the formation of type H vessels and mediate the coupling of angiogenesis and osteogenesis.

In recent years, mechanotransduction pathways have been revealed as key molecular mechanisms regulating macrophage-mediated coupling of H-type angiogenesis and osteogenesis. Among these, Piezo1, a mechanosensitive ion channel highly expressed in macrophages, is capable of converting mechanical stimuli into biochemical signals. According to a study by Liu *et al*., mechanical loading promotes the expansion of F4/80^+^ macrophages. Through a Piezo1-dependent mechanism, these macrophages subsequently transform into the F4/80^+^CD206^+^ regenerative subset, thereby markedly enhancing the spatial coupling between type H vessels and osteoprogenitors at the site of bone defects. Conditional knockout of Piezo1 in Lysozyme M-positive myeloid cells attenuated the aforementioned effects induced by mechanical loading. Furthermore, implantation of wild-type bone marrow-derived macrophages (BMDMs) into the defect area of Piezo1-knockout mice restored mechanosensitive angiogenesis-osteogenesis coupling and promoted bone regeneration [67]. This suggests that Piezo1 mediates mechanosensitive angiogenic-osteogenic coupling in macrophages, serving as a crucial molecular switch linking the mechanical microenvironment to bone repair.

Another important pathway involves Yes-associated protein 1 (YAP1). As a downstream effector of the Hippo pathway, YAP1 exerts dual regulatory effects on both angiogenesis and osteogenesis in macrophages. M2c macrophages can dual-regulate angiogenesis and osteogenesis through the secretion of YAP1. On one hand, YAP1 upregulates the expression of VEGF and Angiopoietin-1 (ANG1) in human umbilical vein endothelial cells (HUVECs), enhancing EC proliferation, migration and tube formation capacity. On the other hand, YAP1 activates the Hippo pathway, promoting the osteogenic differentiation of BMSCs and increasing alkaline phosphatase (ALP) activity, calcium nodule formation and the expression of osteocalcin (OCN) and osteopontin (OPN) [68]. Of note, there exists functional crosstalk between Piezo1 and YAP1: mechanically activated Piezo1 signaling can regulate the nuclear translocation and transcriptional activity of YAP1, thereby coupling mechanosensing with osteogenic-angiogenic gene expression programs.

More importantly, the significance of the Piezo1 and YAP1 pathways extends far beyond angiogenic–osteogenic coupling. For immunomodulation, Piezo1-mediated macrophage polarization directly determines the timing of inflammation resolution; YAP1 also participates in regulating the transcriptional balance of pro-inflammatory and anti-inflammatory factors in macrophages. For angiogenesis, both pathways synergistically promote the functional maturation of H-type endothelial cells. For osteogenic differentiation, they collectively drive the osteogenic fate of BMSCs through paracrine signaling and intracellular signals. Therefore, Piezo1 and YAP1 function as a molecular hub integrating macrophage-mediated immunity, angiogenesis and osteogenesis, providing precise targets for the design of biomaterials aimed at temporally regulating the osteoimmune microenvironment.

### Stage-dependent role of macrophages in bone defect repair

**Figure 3:**
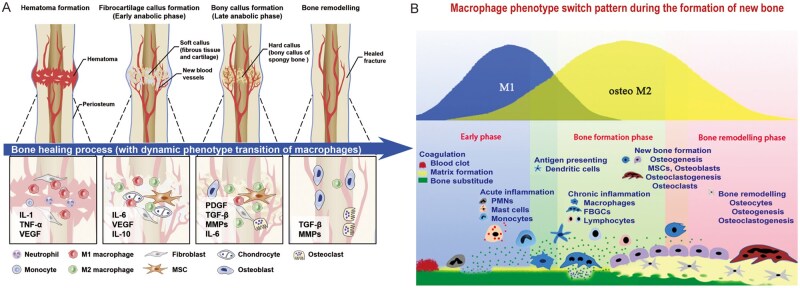
Macrophages in bone defect repair. (**A**) Macrophages participate in the entire process of fracture healing [11]. (**B**) During the progression of bone repair, macrophages undergo dynamic phenotypic and functional transitions, accompanied by the secretion of cytokines that facilitate bone formation [2].

Bone repair is a highly organized and dynamically evolving physiological process, typically consisting of three sequential and partially overlapping phases: the inflammatory phase, the reparative phase and the remodeling phase. As the central regulatory cells of the osteoimmune microenvironment, macrophages persist throughout the entire bone repair process, with their phenotypes and functions dynamically evolving across different repair stages to precisely coordinate the three core events of immune response, vascular remodeling and bone formation [18] (Figure 3).

Within hours to days following bone injury, damage-associated molecular patterns (DAMPs) released from local vascular rupture rapidly activate the innate immune system. As first responders, macrophages extensively infiltrate the defect site within 24–72 h post-injury. Under the stimulation of IFN-γ and LPS, the NF-κB and STAT1 signaling pathways are activated, leading to macrophage polarization toward the M1 phenotype, characterized by high expression of pro-inflammatory cytokines such as TNF-α, IL-1β and IL-6 [31]. During the inflammatory phase, M1 macrophages efficiently clear invading pathogens through phagocytosis and oxidative burst, and secrete various proteases to degrade damaged extracellular matrix debris, thereby creating space for subsequent tissue ingrowth. In addition, M1 macrophages secrete VEGF to initiate the vascular response and recruit MSCs and endothelial progenitor cells via chemokines such as CCL2 and CXCL8, laying the cellular foundation for subsequent angiogenesis and bone formation [69]. A moderate and controlled early inflammatory response positively contributes to subsequent bone regeneration. Zhang *et al*. [70] demonstrated that OPC/LPS composite scaffolds, which initially release LPS to induce M1 polarization, exhibited superior osteogenic effects compared to OPC scaffolds that only promote M2 polarization. According to a survey by Chow *et al*. [71], this M1-dominant phase typically lasts approximately 3–7 days. Schlundt *et al*. [10] found that M1 macrophages are mainly present during the vasodilation phase on days 1–3 post-operation, whereas M2 macrophages gradually become dominant during the vasodilation phase and the subsequent vascular remodeling phase; by Day 7, the inflammatory phase gradually subsides, and M2 macrophages replace M1 macrophages as the dominant population.

As the inflammatory response gradually subsides, negative feedback regulators such as IL-10 secreted by M1 macrophages begin to accumulate, and the concentrations of Th2-type cytokines including IL-4 and IL-13 from apoptotic neutrophils and local stromal cells increase. These signals activate the STAT6 and PPAR-γ pathways, promoting the transition of macrophages toward the M2 phenotype and initiating inflammation resolution and tissue repair programs [32]. Angiogenesis alleviates hypoxic conditions, downregulates the expression of glycolytic enzymes and restores oxidative phosphorylation (OXPHOS), further facilitating the M1-to-M2 transition [72]. Upon entering the reparative phase, M2 macrophages gradually become the dominant population. They suppress residual inflammation by secreting anti-inflammatory cytokines such as IL-10 and TGF-β, and induce ECs proliferation and lumen formation by secreting pro-angiogenic factors including VEGF and PDGF-BB. Furthermore, M2 macrophages secrete pro-osteogenic factors such as BMP-2 and IGF-1, which activate key osteogenic transcription factors including Runx2 and Osterix in MSCs, driving their differentiation toward osteoblasts.

During the late reparative and remodeling phases (beyond 14 days post-injury), M2 macrophages persistently reside within the callus and newly formed bone tissue. On one hand, macrophages assist in maintaining local vascular homeostasis by continuously secreting bioactive molecules such as TGF-β, VEGF and PDGF-BB, supporting terminal differentiation of osteoblasts and bone matrix mineralization. On the other hand, macrophages precisely control the recruitment and activation of osteoclasts by modulating the RANKL/OPG ratio, ensuring functional adaptive remodeling of newly formed bone tissue in response to mechanical demands. Furthermore, macrophages engulf apoptotic chondrocytes through efferocytosis, a process that activates the PI3K/AKT pathway via MSR1 and upregulates PGC1α expression, thereby enhancing mitochondrial OXPHOS and promoting M2 polarization, while concomitantly stimulating BMP-7 production to facilitate osteogenic differentiation of BMSCs.

In summary, macrophages exhibit distinct temporal and functional characteristics across different stages of bone repair. During the inflammatory phase, the M1 phenotype predominates, mediating host defense and tissue repair. During the reparative phase, the M1-to-M2 transition orchestrates inflammation resolution, angiogenesis and matrix deposition. During the remodeling phase, M2 subtypes persist, maintaining bone homeostasis and functional adaptation. Macrophages temporally regulate the two core processes of vascular remodeling and bone formation while mediating the intricate coupling between them, ultimately determining the efficiency and quality of bone repair. This temporal-based functional framework of macrophages provides a theoretical foundation for designing bone repair materials that can mimic or actively guide the spatiotemporal sequence of endogenous immune responses.

## Strategies for regulating the sequential polarization of macrophages

Numerous studies have demonstrated that the M1/M2 transition plays a critical regulatory role in bone defect repair. On the one hand, prolonged polarization of M1 macrophages can lead to chronic inflammation, whereas timely M2 polarization promotes inflammation resolution and prevents the occurrence of an inflammatory storm. On the other hand, while M2 macrophages can induce the differentiation of fibro-adipogenic progenitors toward fibroblasts, excessive duration of M2 polarization can result in pathological fibrosis [73]; this process can in turn be suppressed by TNF-α secreted by M1 macrophages [74]. Under normal physiological conditions, the two phenotypes appear sequentially and maintain a dynamic balance. Therefore, regulating the sequential polarization of macrophages toward M1 and M2 phenotypes represents a key immunomodulatory strategy for novel bone repair materials. This approach promotes angiogenesis, new bone formation, and osseointegration at the defect site, thereby achieving robust bone repair.

In studies related to sequential macrophage polarization, cytokines are the most frequently used regulatory factors, including LPS, IFN, IL-4 and IL-10, which directly modulate macrophage polarization phenotypes, as well as monocyte chemoattractant protein-1 (MCP-1), which recruits macrophages to the injury site in response to the inflammatory microenvironment. Bioactive ions are also widely applied; inorganic ions such as Li^+^ [75], Cu^2+^ [76], Zn^2+^ [5], Mn^2+^ [77], Mg^2+^ [78], Sr^2+^ [79] can regulate the polarization state of macrophages and modulate the osteoimmune microenvironment, thereby improving tissue regeneration outcomes. In addition, numerous natural compounds, such as curcumin [80, 81], quercetin [7], cinnamaldehyde [82] and tannic acid [83], can exert their effects by modulating key signaling pathways, while microRNAs can also mediate macrophage immunomodulation [84, 85]. Delivery systems that transport these cytokines and bioactive ions can achieve sequential modulation of macrophages by influencing important signaling pathways such as NF-κB, MAPK, PI3K-Akt, JAK-STAT, NLRP3, Notch and HIF-1 [86]. Currently, the main delivery strategies in bone tissue engineering can be summarized into three categories: controlled release mechanisms, spatial engineering of carriers and stimuli-responsive design [87]; specific examples are presented in Table 1.

**Table 1:** Design strategies for bone repair materials with the function of regulating sequential macrophage polarization.

Strategies	Biomaterials	Regulatory factors		References
		Early M1 polarization	Late M2 polarization	
		The approach to achieve sequential release		
**Release mechanisms**	Decellularized bone scaffold	IFN-γ	IL-4	[88]
		Physical adsorption	Mediation by biotin-streptavidin binding	
	Nanofiber membrane	Cu^2+^	Zn^2+^	[89]
		Swelling of gelatin membrane	Degradation of PCL membrane	
	CaSiO_3_-β-TCP scaffolds	IFN-γ	Si^4+^	[90]
		Physical adsorption	Degradation of CaSiO_3_ scaffold	
	Ti substrate	LPS	Mg^2+^, nanostructures and wetting angle	[91]
		Adsorption and ions exchange	Ion exchange and crystal structure destruction	
	SIS membrane with SrHA coatings	IFN-γ	Sr^2+^	[92]
		Physical adsorption	Degradation of SrHA coatings	
	Hydroxyapatite/PCL/PLGA Composite Scafflod	LPS	Sr^2+^	[93]
		Physical adsorption	Degradation of scaffold	
**Spatial structure**	PEEK	Cu^2+^	Sr^2+^	[94]
		Outer layer and inner layer of BGN core-shell structure		
	Composite hydrogel of engineered protein and sodium alginate	Ca^2+^	Zn^2+^	[95]
		Outer layer and inner layer of ZPHs core-shell structure		
	dECM	Inflammatory cytokines secreted by M1	Pro-healing cytokines secreted by M2	[96]
		Outer layer and inner layer of concentric cylinder structure		
	Polystyrene disk	IFN-γ	Simvastatin	[97]
		Surface layer and inner layer of multilayer structure via coating strategies		
	Ti nanotubes	IFN-γ	IL-4	[98]
		Entrapped between two gel layers with different degradation rates	Loaded in TNT	
	Multidomain peptides	MCP-1	IL-4	[99]
		Different interactions between MDP and drugs		
**Responsiveness to stimuli**	Porous gelatin two-compartment system	MCP-1 and IFN-γ	IL-4 and IL-10	[100]
		Outer layer and inner layer capable of undergoing magnetic responsive deformation		
	dECM cryogel	CPN	dECM	[101]
		Photothermal-responsive hydrogel systems undergo phase transition	Degradation exposes its active sites	
	Composite hydrogel of gelatin and PEG-benzaldehyde	ZnO	AVs	[102]
		Responsive release under acidic conditions	Degradation of Hydrogel network	
	Shape memory films	Flat surface	Microgrooved surface	[103]
		Morphological transformation in response to near-infrared radiation		

### Release mechanisms

The core of this strategy lies in achieving sequential delivery of M1 and M2 polarization signals over time by modulating the release kinetics of drugs/bioactive factors, thereby matching the natural transition of macrophages from a pro-inflammatory to a pro-reparative phenotype during bone repair. Specifically, drug release can be mediated by various factors, including passive diffusion, carrier swelling/degradation and affinity interactions. By selecting and combining different release mechanisms, the rate and timing of drug release can be controlled, thereby enabling sequential polarization of macrophages.

Driven by the concentration gradient between different regions, drugs can be released from carrier materials by passive diffusion, representing a simple and effective drug release strategy. However, drug diffusion is influenced by factors like the molecular structure and carrier material porosity, as well as the intrinsic properties of the drug itself, resulting in drawbacks including uncontrolled release kinetics and a lack of precise drug targeting. In carrier swelling or degradation-mediated drug release, therapeutic agents are encapsulated within a matrix system. Water infiltration leads to relaxation of the polymer chain network, inducing material swelling or progressive disintegration, which subsequently facilitates drug dissolution and release. Compared with passive diffusion, this strategy typically enables controlled and predictable drug release kinetics by modulating carrier properties such as solubility and degradation rate [104]. For affinity-mediated drug release, the process relies on ligand-receptor interactions that connect the pharmacological agent to the delivery vehicle and encompass hydrophobic effects, van der Waals attractions, hydrogen bonds and electrostatic forces [105]. By specific ligand modification of the carrier material, precise control of drug release rates can be achieved, even enabling responsive drug release and targeted delivery.

**Figure 4:**
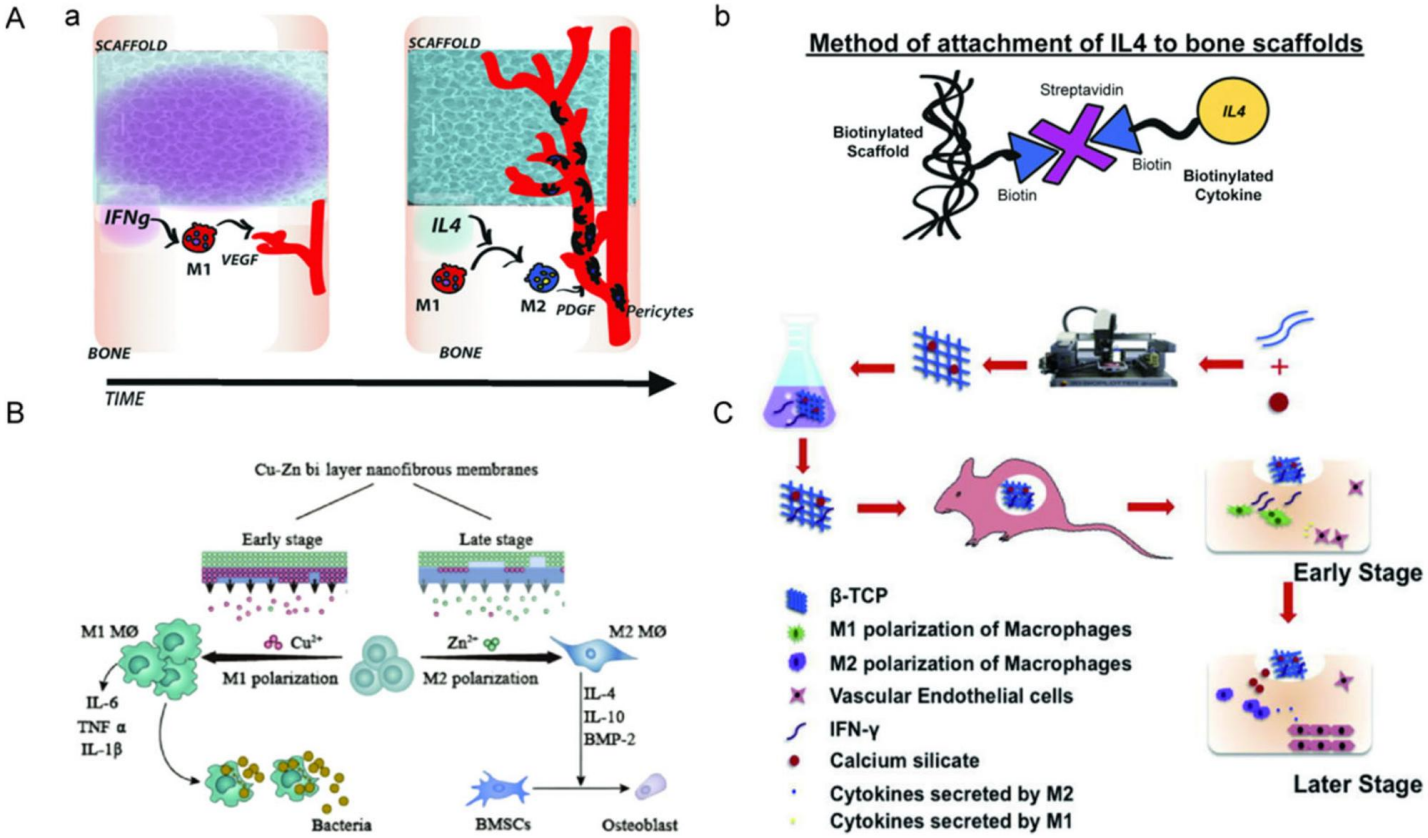
Design of drug release mechanisms. (**A**) Sequential release of IFN-γ and IL-4, with IFN-γ being physically adsorbed onto the scaffold (a), and the release of IL-4 is mediated through affinity (b) [88]. (**B**) The gelatin membrane releases Cu^2+^ upon swelling, while the PCL membrane releases Zn^2+^ upon degradation [89]. (**C**) Physically adsorbed IFN-γ is rapidly released, whereas Si^4+^ is slowly released as the scaffold degrades [90].

Spiller *et al*. [88] physically adsorbed IFN-γ onto decellularized bone-based scaffolds, and subsequently biotinylated both IL-4 and the scaffolds, followed by conjugation via streptavidin. Given that physical adsorption relies on relatively weak non-covalent interactions, whereas the streptavidin–biotin interaction is among the strongest known non-covalent bonds, this strategy achieved a sequential release profile characterized by the initial release of IFN-γ followed by the later release of IL-4 (Figure 4A). This work demonstrated, for the first time, precise control over sequential macrophage polarization in osteoimmunomodulatory scaffolds. Zhao *et al*. developed a multifunctional titanium surface coating composed of Cu_2_O and the E7-KR12 peptide. Specifically, a Cu_2_O layer was deposited onto the polydopamine (PDA)-coated titanium surface via electrostatic adsorption, and the E7-KR12 peptide was subsequently grafted onto the surface through chemical crosslinking. In the early stage, Cu_2_O provided bactericidal activity and promoted M1-type macrophage polarization. In the later stage, the released KR12 from the E7-KR12 peptide offered sustained antimicrobial activity, while the E7 component facilitated the recruitment of BMSCs and promoted M2 polarization [106].

Furthermore, leveraging differential rates of carrier swelling and degradation for drug release enables the regulation of sequential macrophage polarization by biomaterials. Guo *et al*. fabricated a copper-loaded gelatin membrane and a zinc-loaded polycaprolactone (PCL) membrane via electrospinning, followed by thermal pressing to assemble a Cu-Zn bilayer nanofibrous membrane (Figure 4B). Owing to the relatively hydrophilic nature of gelatin, which undergoes rapid swelling/degradation in physiological environments, the upper layer enabled fast release of Cu^2+^. In contrast, PCL, being a hydrophobic polyester with slow degradation kinetics, resulted in delayed release of Zn^2+^ from the lower layer [89]. Zhang *et al*. encapsulated LPS in GelMA hydrogels, which were then embedded into 3D-printed osteogenic peptide-coated poly(L-lactic acid) (OPC/PLLA) scaffolds. This design leveraged the rapid degradation of the hydrogel to release LPS, while the slow degradation of the PLLA matrix enabled the release of OPC [70].

Leveraging non-covalent interactions to achieve the rapid early release of M1-inducing factors, combined with the gradual degradation of carrier materials to enable sustained late release of M2-inducing factors, represents a commonly employed design strategy for achieving sequential macrophage polarization. Li *et al*. prepared a calcium silicate/β-tricalcium phosphate (CaSiO_3_-β-TCP) scaffold by 3D printing (Figure 4C). IFN-γ was loaded via the same physical adsorption method and released quickly in the first 48 h, followed by the swelling and degradation of the scaffolds, leading to gradual release of Si^4+^. This resulted in sequential polarization of M1 and M2 macrophages [90]. Similarly, Liang *et al*. incorporated LPS into Mg-Fe layered double hydroxide (LDH) coatings via electrostatic interactions. The competitive exchange of LPS with interlayer anions of LDH enabled rapid LPS release, while the sustained degradation of the LDH layered structure facilitated the continuous slow release of Mg^2+^ [91]. Yang *et al*. introduced a strontium-substituted nano-hydroxyapatite coating and IFN-γ onto the surface of native small intestinal submucosa (SIS) membranes. Physically adsorbed IFN-γ achieved rapid early release, whereas Sr^2+^ was continuously released as the hydroxyapatite degraded [92]. In addition to the above examples, this strategy has been extensively validated across various material platforms [93, 107].

Among strategies that modulate sequential macrophage regulation by altering release mechanisms, the design logic is relatively straightforward. By exploiting differences in release kinetics, including physical adsorption, affinity binding or carrier degradation, the sequential delivery of M1 and M2 polarization factors can be achieved. These strategies are compatible with various carrier formats, including scaffolds, hydrogels and coatings, and involve relatively mature fabrication processes. However, the limitations of this approach are also significant. For instance, the release sequence is predetermined by the intrinsic properties of the material and cannot be dynamically adjusted based on individual patient differences or pathological states, thus lacking clinical adaptability. Moreover, the release profiles of the two factors often exhibit overlap or ‘tailing’ phenomena, making it difficult to achieve precise temporal switching and potentially causing the M1/M2 transition window to deviate from the optimal range. Therefore, although release mechanism-based strategies are widely used in fundamental research, their translation to complex clinical scenarios requires synergistic optimization with spatial engineering strategies or stimulus-responsive approaches.

### Carrier spatial structure

In addition to drug release mechanisms, the spatial architecture of carriers can also be exploited to design sequential drug release behavior. This strategy achieves differential drug release rates between regions by physically segregating distinct regulatory factors into specific zones of the carrier, leveraging steric hindrance. Common approaches include core-shell structures, concentric bilayer cylindrical structures, multilayer parallel structures and composite architectures.

**Figure 5:**
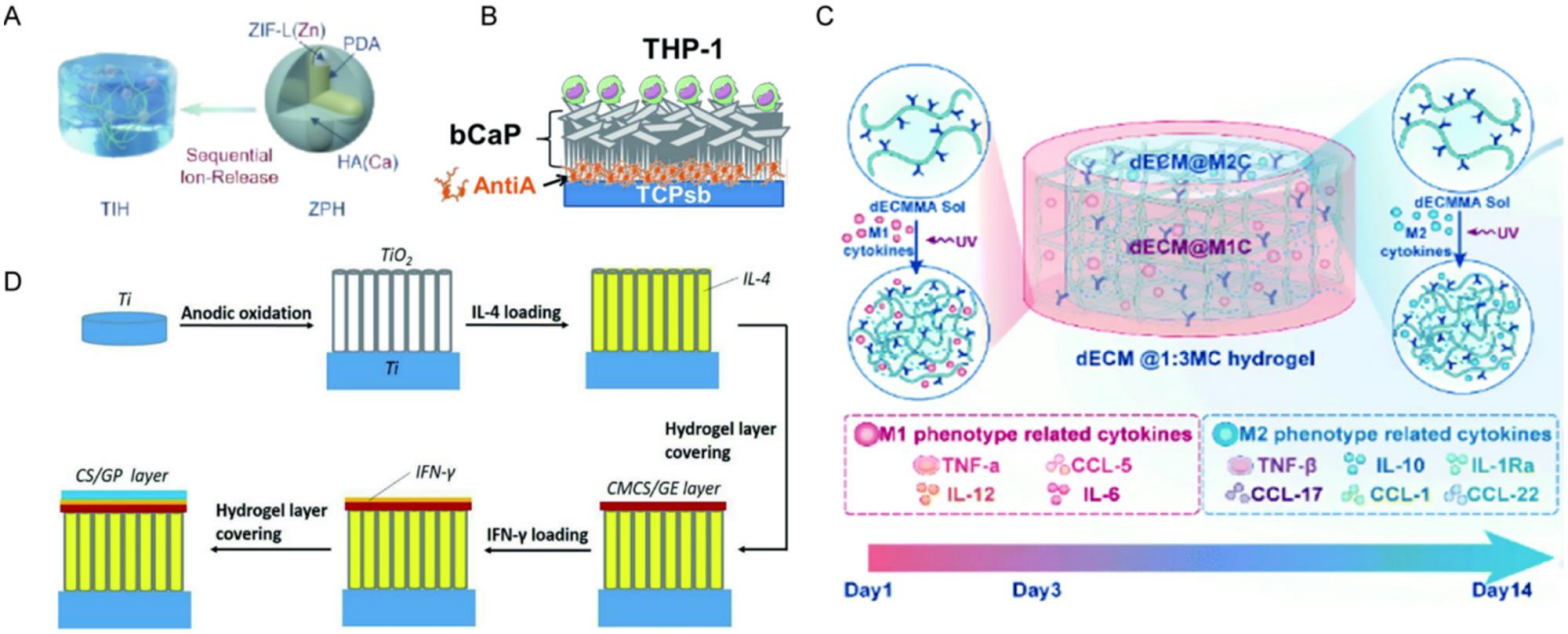
Spatial structure designs for sequential drug release. (**A**) A core-shell structure is utilized to achieve the sequential release of inducing factors [95]. (**B**) A coating is constructed to provide a spatial barrier [97]. (**C**) Drugs are encapsulated within a concentric bilayer cylindrical configuration to achieve sequential release [96]. (**D**) Drugs are encapsulated within a parallel-layer structure to enable sequential release [98].

The core-shell structure is one of the most commonly employed strategies for constructing spatially programmed sequential delivery carriers. Upon implantation, the shell layer, which is directly exposed to the microenvironment, degrades rapidly and releases the encapsulated cargo, achieving a high local concentration for prompt intervention. In contrast, the release of factors from the core is restricted due to the physical barrier provided by the shell layer. After degradation of the shell, the core becomes exposed and releases the internal factors in a sustained manner, thereby enabling the programmed sequential release of bioactive factors. Wu *et al*. constructed a unique core-shell structure in which Cu^2+^ and Sr^2+^ were sequentially incorporated into bilayered bioactive glass nanoparticles (BGNs). These ions were chelated onto the surface of sulfonated polyetheretherketone (PEEK) via PDA. Cu^2+^ and Sr^2+^, distributed in the outer shell and inner core, respectively, were released sequentially to achieve sequential macrophage polarization, thereby exerting early-stage antimicrobial and anti-infection functions followed by the promotion of osseointegration and bone repair [94]. Jin *et al*. incorporated zinc/calcium phosphate hybrid nanoparticles (ZPHs) with a core-shell structure into a hydrogel matrix (Figure 5A). Upon release into the bone defect microenvironment, the Ca^2+^ localized in the shell was preferentially released, inducing macrophage polarization toward the M1 phenotype to suppress infection. As the shell degraded, Zn^2+^ from the core was continuously released, subsequently inducing macrophage polarization toward the M2 phenotype and ultimately promoting osteogenic differentiation [95].

Similar to the core-shell structure, the concentric bilayer cylindrical structure also spatially segregates two polarization-inducing factors into an outer layer and an inner core (Figure 5C). Xiao *et al*. fabricated a concentric bilayer decellularized extracellular matrix (dECM), in which the outer and inner layers were composed of pro-inflammatory cytokines secreted by M1 macrophages and pro-healing cytokines secreted by M2 macrophages, respectively. The spatiotemporal cascade of M1-to-M2 cytokines enabled immunomodulation throughout the entire functional process of macrophages [96]. In addition to core-shell structures, coating strategies offer a relatively simple approach for spatial separation of two drugs, and the release time and rate of different drugs can be modulated by factors such as coating composition and thickness (Figure 5B). Alhamdi *et al*. sequentially loaded an M2-promoting molecule (simvastatin) and an M1-promoting molecule (IFN-γ) onto the same side of a polystyrene disk, with a biomimetic calcium phosphate (bCaP) layer serving as a physical barrier. This spatially layered structure achieved physical separation of the two molecules with opposing polarization functions. In the early stage of implantation, the superficial IFN-γ layer was preferentially exposed and rapidly released, inducing M1 polarization. In contrast, the underlying simvastatin exhibited delayed release due to the barrier effect of the bCaP coating, becoming accessible only after degradation or dissolution of the coating, thereby achieving subsequent induction of M2 polarization [97].

Multilayer parallel structures can also spatially segregate polarization-inducing factors (Figure 5D). Chen *et al.* achieved sequential release of IL-4 and IFN-γ by constructing a composite spatial architecture integrating titanium nanotubes (TNTs) with a bilayer hydrogel. Specifically, IL-4 was loaded into the TNT reservoirs, which were subsequently covered with a chemically crosslinked lower hydrogel layer composed of carboxymethyl chitosan and genipin (CMCS/GE) to serve as a sealing and sustained-release barrier for IL-4. IFN-γ was then adsorbed onto this hydrogel layer and encapsulated within an upper hydrogel layer formed by physically crosslinked chitosan and β-glycerophosphate disodium (CS/GP), thereby establishing a multilayered spatial structure. Owing to the relatively weak stability of the physically crosslinked hydrogel, IFN-γ was rapidly released, whereas the dense structure of the chemically crosslinked hydrogel delayed the release of IL-4. Notably, this strategy does not rely solely on spatial configuration but rather operates synergistically with release mechanisms, exhibiting superior advantages in fabrication tunability and precise temporal control [98].

In addition to the basic strategy of setting up steric hindrance to distribute different drugs in different regions, special spatial structures have been designed in some studies for the purpose of immunomodulation of sequential polarization of macrophages. Multidomain peptides (MDPs) are highly efficient drug carriers capable of forming nanofiber hydrogels through β-sheet folding. By adjusting the interactions between MDPs and the loaded drugs, the release profiles of the drugs can be customized. For instance, Kumar *et al*. developed advanced MDPs designed to achieve a fast release of MCP-1, facilitating monocyte recruitment during the early inflammatory phase. This was subsequently followed by a prolonged release of IL-4, which promoted the polarization of macrophages toward the M2 phenotype. The resulting immune modulation was found to be effective under both *in vitro* and *in vivo* conditions [99].

In spatial engineering strategies, various types of factors, such as cytokines, ions and nanoparticles, can be accommodated, avoiding interference among different components. Parameters including coating thickness and crosslinking density can be flexibly tuned to meet diverse temporal requirements. However, the limitations of this strategy are also evident. The fabrication processes for multilayer or core-shell structures are complex, require high precision, and pose challenges in maintaining batch-to-batch consistency. The interfacial bonding strength between different material layers may be inadequate, presenting risks of delamination or detachment. Furthermore, after the release of outer-layer factors, the prolonged diffusion path of inner-layer factors may result in delayed release, thereby compromising the precision of the M1/M2 transition. Therefore, spatial engineering strategies require further optimization in structural design and interface engineering to enhance their reliability and regulatory precision.

### Responsiveness to external stimuli

Designing osteoimmunomodulatory materials that respond to external physical stimuli (e.g. light, ultrasound, magnetic field, temperature) and chemical stimuli (e.g. pH) to mediate the sequential transition of regulatory factors represents an intelligent strategy for modulating macrophage polarization. Unlike the release mechanisms discussed earlier, external stimulus-responsive strategies endow implantable materials with on-demand regulatory capabilities, enabling precise control over both the timing and duration of immunomodulatory factor release. Therefore, intelligent responsive designs allow for flexible modulation based on individual patient differences and pathological conditions.

**Figure 6:**
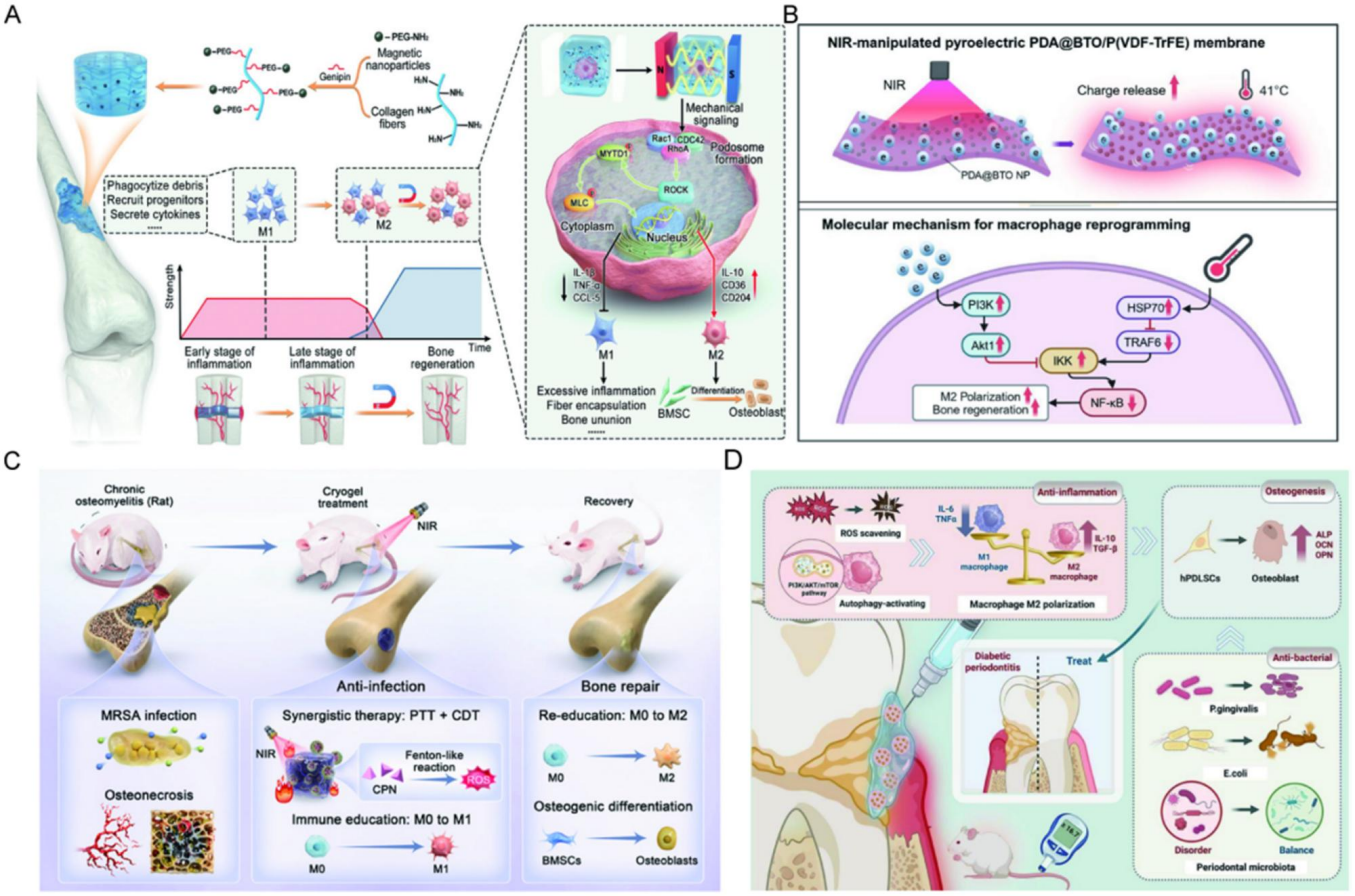
Design of responsiveness to stimuli. (**A**) Magnetoresponsive materials enable the temporal progression of macrophage polarization under the regulation of an external magnetic field [108]. (**B**) Photothermal effect and charge signals synergistically regulate macrophage polarization [109]. (**C**) Photothermal therapy and photodynamic therapy achieve bidirectional regulation of macrophages through thermal stress and oxidative stress [101]. (**D**) pH/ROS dual-responsive hydrogels modulate macrophage polarization [110].

Based on the aforementioned design concepts, Tolouei *et al*. [100] developed a magnetoresponsive dual-chamber system to address the need for flexible control over inflammation duration. The system consists of an outer chamber made of porous gelatin and an inner chamber composed of a deformable columnar gel. The outer chamber enables rapid release of pro-inflammatory factors (MCP-1, IFN-γ) via passive diffusion, promptly establishing a pro-inflammatory microenvironment and recruiting macrophages. The inner chamber is divided into an upper region containing Fe_3_O_4_ magnetic particles and a lower region containing anti-inflammatory factors such as IL-4 and IL-10. Upon exposure to an external magnetic field, the inner chamber deforms, leading to the release of anti-inflammatory factors. In this manner, the timing of the M1-to-M2 macrophage transition can be controlled by regulating the application time of the external magnetic field. Extending this magnetoresponsive regulation strategy, Huang *et al*. grafted superparamagnetic nanoparticles onto collagen nanofibers to construct a superparamagnetic hydrogel capable of temporally regulating macrophage polarization (Figure 6A). By applying a static magnetic field in a time-delayed manner, a temporally engineered M1-to-M2 transition process was established [108].

In addition to magnetoresponsive systems, other external stimulus-responsive platforms have also demonstrated potential in the temporal regulation of macrophages. Guo *et al*. developed a near-infrared (NIR) remotely controllable dynamic regulation strategy, in which PDA-modified barium titanate nanoparticles were incorporated into a piezoelectric polymer to fabricate composite films (Figure 6B). NIR irradiation elevated the material temperature to 41°C while enabling on-demand charge release via the pyroelectric effect. During bone healing, this strategy promoted M1 polarization within the initial 1–2 days and induced M2 switching by day 7, synergistically regulating macrophage phenotype through the HSP70/AKT-NF-κB pathway under combined thermal and electrical stimulation, thereby achieving sequential polarization [109]. Yu *et al*. designed a bidirectional delivery system composed of graphene oxide (GO) and urolithin A (UA). Upon NIR irradiation, the system promoted M1 polarization of macrophages and enhanced their phagocytic activity via the Integrin-RhoA-ROCK1 pathway. Subsequently, as the substrate degraded, UA was sustainably released, inducing phenotype switching and effectively preventing infection while promoting bone regeneration [111]. The dual-stimuli-responsive cryogel designed by Liu *et al*. also achieved sequential regulation of macrophages (Figure 6C). Under NIR irradiation, the material exhibited a photothermal effect. When the temperature exceeded 45.4°C, the loaded copper peroxide nanoparticles (CPN) were released, eliminating infecting bacteria while promoting M1 macrophage polarization. Subsequently, components such as dECM within the cryogel induced macrophage transition toward the M2 phenotype, creating a supportive microenvironment for bone regeneration [101].

Harnessing the characteristic signals of the bone defect microenvironment as endogenous switches also enables the sequential release of programmed polarization-inducing signals. Liu *et al*. incorporated ZnO into a hydrogel matrix via Schiff base reaction and Zn^2+^-catechol chelation, along with loaded apoptotic vesicles (AVs). In response to the acidic conditions of infected bone defects, ZnO nanoparticles were continuously released, promoting M1 polarization while activating phagocytic function to enhance AVs delivery efficiency. Subsequently, the pro-inflammatory M1 microenvironment was reversed to induce M2 polarization [102]. Distinct from the conventional passive M1–M2 switching mode, this design achieves active regulation through functional coupling among components, effectively mitigating the risk of chronic inflammation resulting from prolonged M1 duration. In response to the pathological features of low pH and high reactive oxygen species (ROS) levels in the diabetic bone defect microenvironment, Ai *et al*. [110] developed a pH/ROS dual-responsive hydrogel. This system incorporated ROS-responsive boronic ester linkages as sensing moieties, while utilizing acid-labile organic ligands of the ZIF-8 metal-organic framework to enable structural dissociation and subsequent release of encapsulated drugs under acidic microenvironmental conditions (Figure 6D). This endogenous stimulus-responsive strategy based on microenvironmental characteristic signals enables adaptive recognition of pathological states and on-demand drug delivery, demonstrating substantial application potential in the treatment of bone defects under specific pathological backgrounds such as diabetes mellitus.

Compared with release mechanism-based strategies and spatial engineering strategies, stimulus-responsive strategies offer the advantages of high regulatory flexibility, real-time decision-making on release timing by the operator and dynamic adjustability according to individual patient differences or pathological conditions. Moreover, external stimuli enable on-off release, thereby circumventing the issues of curve overlap or tailing commonly observed in release mechanism-based approaches. These features confer unique advantages in complex pathological scenarios such as diabetes and infection. Nevertheless, this strategy also has certain limitations, including the limited penetration depth of stimuli such as near-infrared light in deep tissues, as well as the increased fabrication complexity and safety costs associated with the incorporation of responsive components. Future research should focus on developing responsive material systems that combine deep tissue penetration capability with biosafety to facilitate the clinical translation of this strategy.

### Immunomodulatory functions of physicochemical properties of materials

Current temporal polarization strategies heavily rely on the delivery of exogenous immunomodulatory factors, while insufficient attention has been paid to the role of the physicochemical characteristics of the material itself in regulating macrophage polarization. In fact, the intrinsic properties of materials, such as chemical composition, stiffness, topography, viscoelasticity, porosity and hydrophilicity/hydrophobicity, can also significantly influence the polarization direction and functional phenotype of macrophages [14, 16, 112, 113]. Moreover, compared with bioactive factors, these properties offer superior stability and safety.

As the most fundamental physicochemical property of materials, chemical composition regulates macrophage adhesion, polarization and functional phenotype, serving as a key parameter in the design of osteoimmunomodulatory materials. The immunomodulatory functions of the intrinsic chemical composition of materials have been widely demonstrated. For instance, hydrogel systems based on type I collagen and chitosan promote M2-type polarization of macrophages without the addition of exogenous cytokines, significantly improving angiogenesis and alleviating chronic inflammation in diabetic wounds [114]. Chondroitin sulfate has also been shown to effectively induce M2 phenotypic transformation [115]. Chemical modification of materials enables tailored regulation of their immunomodulatory properties. For example, dicyandiamide-modified chitosan tends to induce M1-type macrophage polarization, whereas polyethylene glycol (PEG)-modified chitosan promotes M2-type polarization [116]. Hydrogel systems composed of methacryloylated gelatin and nitrobenzyl-modified gelatin have been shown to effectively promote M2 macrophage polarization, alleviate inflammation and reduce fibrosis [117]. Based on these insights, the inherent immunomodulatory properties of materials can be leveraged as fundamental signals in the design of temporally programmed osteoimmunomodulatory materials. Wan *et al*. [118] developed a Cu^2+^/anthocyanin composite coating system, in which the released Cu^2+^ induces macrophage polarization toward the pro-inflammatory M1 phenotype at the early stage, whereas as the release rate of Cu^2+^ diminishes, the *in situ* immobilized anthocyanins gradually become the dominant signal, driving macrophage switching toward the anti-inflammatory M2 phenotype. Huang *et al*. demonstrated that the magnetoelectric bioactivity of transition metal carbides/nitrides (MXene)-based nanosheets enables precise regulation of macrophage polarization. Under an applied rotating magnetic field, MXene generates electrical signals and ROS to induce M1-type polarization; upon removal of the magnetic field, the intrinsic bioactivity of MXene induces M2-type polarization [119].

Matrix stiffness is one of the most thoroughly investigated physical cues regulating macrophage phenotype. Chen *et al.* prepared polyacrylamide hydrogels with varying substrate stiffness and found that at a relatively low substrate stiffness (2.55 ± 0.32 kPa), macrophages cultured on the hydrogel surface exhibited enhanced CD86 expression, indicating polarization toward the M1 phenotype. In contrast, on hydrogels with moderate stiffness (34.88 ± 4.22 kPa), macrophages were more prone to polarize toward the M2 phenotype, with increased CD206 expression and elevated levels of IL-4 and TGF-β [120]. Zhang *et al*. [121] also reported that when macrophages were cultured on collagen-coated polyacrylamide hydrogels, high-stiffness hydrogels (295 kPa) guided macrophages toward the pro-inflammatory M1 phenotype, whereas lower stiffness (18 and 76 kPa) promoted the anti-inflammatory M2 phenotype. Guan *et al*. designed a collagen-based hydrogel that modulated extracellular matrix stiffness through stepwise induction of hydrogel crosslinking, providing a dynamically stiffening 3D culture environment for macrophages. They found that an increase in stiffness alone was sufficient to promote macrophage polarization, mimicking the effect of IL-4 in a soft matrix [122]. However, the relationship between stiffness and macrophage phenotype is not unequivocal. He *et al*. [123] cultured macrophages using transglutaminase-crosslinked gelatin and observed that under low matrix stiffness (1.58 ± 0.42 kPa), the expression of M2 markers IL-10 and Arg increased, whereas under moderate-to-high matrix stiffness, the expression levels of iNOS and TNF-α in macrophages were significantly elevated. It is generally recognized that macrophages are more prone to polarize toward the pro-regenerative M2 phenotype under intermediate matrix stiffness, whereas excessively high or low stiffness tends to maintain the M1 phenotype. Therefore, constructing scaffold systems with dynamically tunable stiffness holds promise for achieving spatiotemporal sequential regulation of macrophage phenotypes.

Surface topography regulates macrophage morphology and function through contact guidance mechanisms [124]. McWhorter *et al*. [125] demonstrated that M1 and M2 macrophages exhibit round and elongated morphologies, respectively, and that narrow microgroove structures can induce macrophage transition toward the elongated M2 phenotype. Leveraging this finding, Zheng *et al*. combined PCL and PEG-modified gold nanorods to fabricate a shape memory film, the surface of which could undergo dynamic changes from flat to microgrooved upon NIR irradiation. This transition induced a morphological change of macrophages toward an elongated shape, subsequently driving a shift from the M1 phenotype to the M2 phenotype characterized by upregulated expression of arginase-1 and IL-10. The effectiveness of this sequential regulation was also validated *in vivo* [126]. In addition to microgrooves, other topographical features have also demonstrated significant immunomodulatory functions. Zhu *et al*. fabricated four types of honeycomb-like titanium dioxide structures with different dimensions on titanium substrates and found that as the structure size decreased, the RhoA/Rho-associated protein kinase signaling pathway was activated, significantly promoting macrophage polarization toward the M2 phenotype. Among these structures, the smallest size (90 nm) exhibited the strongest polarization-inducing capability [127]. Tang *et al*. [128] prepared electrospun PLLA membranes with different configurations and sizes and reported that oriented electrospun membranes with a diameter of 600 nm promoted macrophage polarization toward the M2 phenotype. Luo *et al*. developed a hydrogel with a dynamic RGD-patterned surface via photopatterning technology and the specific interaction between cyclodextrin and azobenzene-modified RGD. Under ultraviolet irradiation, this system enabled on-demand regulation of macrophage morphological transition and sequential polarization [108]. Therefore, by designing dynamically switchable topographical features, static topographical cues can be upgraded into temporally controllable dynamic signals, providing a novel design strategy for constructing sequential polarization strategies.

The Gaussian curvature of material surfaces can serve as a biophysical signal enabling precise regulation of macrophage polarization. Lu *et al*. [129] developed a high-throughput screening chip based on β-TCP and found that negative Gaussian curvature promotes M2 polarization of macrophages by inhibiting the Ras-mitogen-activated protein kinase signaling pathway and downregulating the expression of HIF-1α. The porous structure of scaffolds also significantly influences macrophage phenotype. Koyal Garg *et al*. fabricated scaffolds with varying pore sizes and porosities by adjusting polymer concentration and observed that as fiber diameter and pore size increased, the expression of the M2 marker Arg-1 was upregulated, while the expression of the M1 marker iNOS correspondingly decreased. Notably, pore size was identified as a more critical structural parameter than fiber diameter in regulating macrophage polarization [130]. Furthermore, surface hydrophilicity/hydrophobicity can modulate the adsorption conformation of specific proteins, selectively activating signaling pathways such as PI3K and NF-κB, thereby influencing macrophage behavior [131]. Similarly, charged surfaces play an important role in regulating macrophage polarization by affecting protein adsorption, cell adhesion, and intracellular signal transduction [132]. These multidimensional physical properties can all serve as immunomodulatory signals, providing a rich set of design parameters for constructing sequential polarization materials that do not rely on exogenous cytokines.

In summary, the physicochemical characteristics of implantable materials enable spatiotemporal regulation of macrophage phenotype: at the spatial level, topographical and porous structures guide cellular behavior; at the temporal level, stiffness adaptation and degradation kinetics match the time course of the immune response. Therefore, the synergistic design integrating intrinsic material properties with exogenous regulatory factors represents a promising direction for constructing spatiotemporally controllable immunomodulatory platforms. However, systematic structure–activity relationship studies are still lacking regarding how the regulatory efficacy of the physicochemical properties of bone implant materials can be harnessed for the sequential regulation of macrophages. Most current research remains at the level of qualitative description, making predictable temporal immunomodulation difficult to achieve. Moreover, the effects of physical and chemical cues on macrophage polarization involve complex interactions. For instance, specific surface topographies may synergize with or antagonize the inductive effects of chemical signals [133]. Current understanding of the mechanisms underlying the synergistic regulation of sequential polarization by these two types of cues remains insufficient, limiting the realization of precise multi-factorial synergistic regulation.

## Clinical translation of sequential polarization strategies

### The bone defect microenvironment under diverse pathological conditions

Notably, the current design of sequentially polarizing modulatory materials is predominantly based on idealized timelines derived from healthy animal models. However, factors such as age, different pathological conditions and local infection status can influence the intensity and progression of the immune response during bone repair [134]. Under different pathological conditions, the bone defect microenvironment exhibits significant heterogeneity in terms of cellular composition, metabolic state and immune response characteristics, and the optimal time window for macrophage transition from the M1 to the M2 phenotype also varies. This discrepancy poses additional challenges to sequential polarization strategies.

#### Diabetic bone defects

Diabetic bone defects represent one of the most formidable clinical challenges in the field of bone repair and regeneration. The hyperglycemic microenvironment can activate the NF-κB and mechanistic target of rapamycin complex 1 signaling pathways, thereby inducing excessive ROS production [135]. Serving as crucial signaling molecules, ROS can promote the polarization of M1 macrophages via two key kinases in the DNA damage response pathway: ataxia-telangiectasia mutated (ATM) and checkpoint kinase 2 (Chk2) [136]. With the prolonged persistence of the M1 phenotype, the expression levels of TNF-α, iNOS and IL-6 are significantly elevated, which subsequently induces the apoptosis of BMSCs and osteoblasts. Furthermore, under high ROS levels, BMSCs suffer from mitochondrial dysfunction, and their responsiveness to osteogenic signals is markedly diminished, ultimately leading to impaired osteogenic differentiation potential [137]. Research data indicate that the fracture healing period in diabetic patients is prolonged by an average of 87% compared to healthy individuals [138].

In response to the polarization imbalance commonly observed in the diabetic bone defect microenvironment, characterized by excessive activation of M1 macrophages and impaired or even absent M2 polarization, the sequential polarization strategy should be conceptualized as a ‘forced reprogramming’ mode, rather than temporal guidance under normal physiological conditions. It is imperative to actively interrupt the continuous input of M1 polarizing signals and significantly enhance both the intensity and duration of M2 polarization-inducing signals to achieve the phenotypic transition of macrophages. Yang *et al*. [139] designed a dynamically cross-linked hydrogel system, which effectively promotes M2 polarization while suppressing M1 polarization through the sustained release of linagliptin. Rather than relying on the passive resolution of M1 signals, this strategy actively downregulates the M1 program and synchronously activates the M2 program, directly reversing the polarization imbalance under diabetic pathological conditions. Xie *et al*. designed a bone-targeted nanoplatform, AgSr-MSNs, which is responsive to the acidic diabetic microenvironment. Under NIR irradiation, this platform guides macrophage polarization toward the M2 phenotype by modulating the JAK/STAT signaling pathway [140].

Furthermore, scavenging ROS and implementing antioxidant interventions can create a favorable environment for M2 macrophage polarization. Li *et al*. fabricated a porous Ti-6Al-4V scaffold modified with a BPQD@MOF composite nanozyme hydrogel. In this system, the BPQD component possesses glucose oxidase (GOx)-like activity to reduce the local glucose concentration, whereas the Cu-MOF exhibits superoxide dismutase-like and catalase-like activities to effectively scavenge ROS and generate O_2_. Through the synergistic dual mechanism of glucose reduction and antioxidation, this self-cascade catalytic system alleviates oxidative stress and improves the local metabolic microenvironment. This restores the responsiveness of macrophages to polarization signals, thereby promoting M2 polarization and the osteogenic differentiation of BMSCs [141]. Similarly, Fu *et al*. designed a glucose/ROS-responsive multifunctional hydrogel. By incorporating GOx and cerium-based nanozymes, this system achieves simultaneous blood glucose reduction and ROS scavenging, consequently inducing the transition of macrophages from the M1 to the M2 phenotype [142].

Notably, the influence of diabetes on macrophage polarization exhibits a ‘metabolic memory’ effect. Even after achieving glycemic control, the epigenetic imprints established by a prolonged hyperglycemic environment in bone marrow macrophage precursors can persist, rendering it difficult for macrophages to fully recover their responsiveness to polarizing signals. Therefore, in parallel with the design of material systems, it is crucial to explore intervention strategies that eliminate the epigenetic imprint of high glucose in macrophages, thereby fundamentally restoring cellular function.

#### Infectious bone defects

In the pathological microenvironment of infectious bone defects (such as osteomyelitis), exogenous pathogens, particularly bacteria, serve as the central triggers for the disruption of host immune homeostasis. Among these, *Staphylococcus aureus* (*S. aureus*) is the predominant pathogen, accounting for over 75% of cases [27]. The inherent paradox in the repair of infectious bone defects lies in the temporal conflict between antibacterial and osteogenic demands: a robust inflammatory response is requisite in the early phase to eradicate pathogens, whereas persistent inflammation is detrimental to bone regeneration. Conversely, the later phase necessitates anti-inflammatory processes and tissue repair, yet premature reparative signals may compromise overall antibacterial efficacy [143].

*S. aureus* disrupts M1 macrophage function through diverse immune evasion mechanisms. These include secreting toxins, such as α-hemolysin and Panton-Valentine leukocidin, to directly lyse macrophages; surviving and disseminating intracellularly via a ‘Trojan horse’ strategy; and forming bacterial biofilms that impair the antimicrobial efficacy of M1 macrophages [27]. In chronic infections, *S.aureus* can further drive the transition of macrophages from an M1-like state to an M2-like phenotype. This phenotypic shift dampens immune clearance and establishes a microenvironment conducive to bacterial persistence and dissemination [144].

Based on disease progression, infections can be categorized into acute and chronic phases. Acute infections are characterized by rapid bacterial proliferation and a robust host immune response. During this stage, macrophages and neutrophils are extensively recruited to the infection site, initiating a strong M1-driven immune response via the secretion of pro-inflammatory cytokines such as TNF-α, IL-1β and IL-6. With timely and effective intervention, this inflammation can resolve naturally. In this context, sequential polarization strategies should be designed as an ‘on-demand switching’ paradigm. This approach prioritizes the eradication of pathogens within the microenvironment by promoting M1 polarization to establish a robust immune defense, thereby preventing bacterial colonization at the defect site. Following infection control, a sequential polarization program is triggered to drive anti-inflammatory and tissue reparative processes. Chronic infections manifest as a vicious cycle of sustained infection and immune dysregulation, wherein macrophages are trapped in a prolonged M1-polarized state, and their transition to the M2 phenotype is severely suppressed. Under these conditions, it is imperative to integrate biofilm-dispersing agents or physical disruption methods. Once the infection is managed, a forced switching strategy must be employed to directly deliver M2-polarizing cues or actively reset the macrophage polarization state.

To treat infectious bone defects, Jin *et al*. [95] developed a sequentially immunomodulatory hydrogel. In this system, Ca^2+^ is rapidly released within 24 h to drive macrophage polarization toward the M1 phenotype via the TRPC1-STAT1/NF-κB pathway, thereby initiating an antibacterial immune response. Subsequently, Zn^2+^ is slowly released over 7 days to induce M2 polarization via the STAT6/PPARγ pathway, promoting osteogenic differentiation. In a rat infected calvarial defect model, the experimental group achieved a bacterial inhibition rate exceeding 90% within 12 h, yielding a significantly higher bone volume fraction compared with the control group. Furthermore, Shen *et al*. designed injectable microspheres comprising an IPN of ionically crosslinked sodium alginate and genipin-crosslinked gelatin, which were further doped with tannic acid and copper ions. Upon NIR irradiation, these microspheres exhibited highly efficient and rapid bactericidal efficacy. They also demonstrated the capacity for sequential macrophage modulation: stimulating M1 polarization during the early stages of osteomyelitis to combat infection, while inducing a phenotypic transition to M2 macrophages in the middle and late stages to facilitate tissue repair [145]. Conversely, stimuli-responsive strategies are better suited for chronic infection scenarios where the optimal switching window is unpredictable. Cui *et al*. developed HAp@MXene composite nanomaterials, which achieved ‘early strong antibacterial effect and later efficient repair’ through programmed immune regulation under the intervention of an external magnetic field *in vitro*. Mechanistically, a rotating magnetic field activates the NF-κB pathway to promote M1 polarization, initiating a pro-inflammatory response to mitigate the risk of infection. Upon removal of the magnetic field, Ca^2+^ released from the material activates the PI3K-Akt pathway, thereby promoting M2 polarization and osteogenic differentiation [146]. This ‘on-off’ switching mechanism enables precise spatiotemporal control over the macrophage polarization state, allowing clinicians to appropriately withdraw the magnetic field based on the infection status to actively trigger the repair program.

Unlike the ‘M1 hyperactivation’ characteristic of diabetic conditions, the core challenge in infectious microenvironments lies in the unpredictability of the polarization switching window. The timeline for successful infection control is contingent upon pathogen virulence, the extent of biofilm formation, and the host immune status. Consequently, pre-programmed temporal release strategies often fail to accommodate inter-individual variability. In the future, it will be necessary to develop biomarkers capable of dynamically monitoring the infection process to enable precise determination of the switching timing. By integrating such biomarkers with intelligent material systems, the temporal window of polarization can be precisely controlled. Only through the dynamic sensing and on-demand regulation of the infection process can sequential polarization strategies achieve their maximum benefit.

#### Post-tumor bone defects

Post-tumor bone defects represent one of the most challenging clinical scenarios in the field of bone tissue regeneration. In primary malignant bone tumors, typified by osteosarcoma, the TME is extensively infiltrated by tumor-associated macrophages (TAMs). Through the secretion of factors such as IL-1β, IL-8 and VEGF, these macrophages drive tumor-associated inflammation, angiogenesis and immune evasion, thereby accelerating the invasion and metastasis of tumor cells [147]. Notably, in stark contrast to the physiological reparative functions of M2 macrophages during normal bone healing, the M2 polarization state of TAMs is fundamentally pathological, essentially functioning to facilitate tumor cell survival and dissemination.

Tumor cells orchestrate the M2 polarization of macrophages and suppress their effector functions through multifaceted mechanisms. On one hand, tumor cells overexpress macrophage colony-stimulating factor (M-CSF), which binds to the colony-stimulating factor 1 receptor (CSF-1R) on the macrophage surface, actively driving M2 phenotypic polarization [148]. On the other hand, tumor cells express the CD47 ‘don’t eat me’ signal, which engages the signal regulatory protein alpha (SIRPα) receptor on macrophages, directly inhibiting their phagocytic capacity [149]. Compounded by the hypoxic, acidic and lactate-rich characteristics of the TME, the M2 polarization state of TAMs is further reinforced, thereby establishing a robust immunosuppressive niche. In this context, sequential polarization strategies should be conceptualized as a ‘bidirectional reprogramming’ paradigm. Initially, it is imperative to reverse the locked M2 state and rapidly mount an M1-dominated anti-tumor immune response, while carefully averting excessively prolonged M1 polarization that could impede bone regeneration. Subsequently, following the eradication of tumor cells, timely induction of M2 polarization is required to initiate the osteogenic repair cascade.

To counteract the M2-locked state of TAMs, researchers have developed multifunctional implantable scaffolds predicated on immune signal blockade. Li *et al*. employed phenylboronic acid-modified mesoporous silica nanoparticles as drug carriers for a CSF-1R inhibitor. Anti-SIRPα antibodies were immobilized on the nanoparticle surface via boron-nitrogen coordination bonds, and this co-delivery system was subsequently loaded into the porous network of a calcium phosphate scaffold utilizing dynamic covalent bonding. By concurrently disrupting both the M-CSF/CSF-1R and CD47/SIRPα signaling pathways, this scaffold effectively reversed M2 polarization, enabling M1 macrophages to dominate the local microenvironment [150]. Similarly, Li *et al*. encapsulated the CSF-1R inhibitor GW3580 within a hydroxybutyl chitosan/oxidized chondroitin sulfate hydrogel coating on the surface of a calcium phosphate scaffold. During the early phase, the released GW3580 blocked the CSF-1R and NF-κB signaling pathways, thereby inhibiting M2 polarization and actively reprogramming M2 macrophages toward the M1 phenotype. This orchestrated the remodeling of the immune microenvironment and robustly suppressed tumor growth. In the subsequent phase, the exposed calcium phosphate scaffold released calcium and phosphate ions to promote the osteogenic differentiation of BMSCs [151].

Another class of strategies focuses on eradicating tumor cells via physical or metabolic interventions. For instance, incorporating Mn_3_O_4_ nanosheets into a gelatin methacryloyl/oxidized dextran double-network hydrogel yields an injectable composite system. In response to the acidic microenvironment, the hydrogel releases Mn_3_O_4_ nanosheets, which target intracellular glucose metabolism and inhibit adenosine triphosphate production, thereby facilitating the ablation of tumor cells under mild hyperthermia (43°C). Subsequently, attenuating the NIR irradiation power enhances osteogenesis [152]. Similarly, a thermosensitive hydrogel encapsulating black phosphorus (BP) nanoparticles can generate localized hyperthermia (45°C–50°C) under NIR irradiation to selectively ablate residual tumor cells. Following tumor eradication, the degradation of BP yields PO_4_^3−^ ions that induce *in situ* calcium phosphate deposition, forming a bone-like apatite layer to promote the osteogenic differentiation of BMSCs [153]. Although the above methods can eliminate tumor cells and promote bone defect repair following tumor resection, none of them incorporate a macrophage polarization regulation module. This makes it difficult to reverse the pathological polarization state of TAMs, which may subsequently compromise bone repair outcomes or even increase the risk of tumor recurrence.

In contrast, the MgO_2_/PLGA composite scaffold fabricated by Li *et al*. via low-temperature 3D printing demonstrates a more comprehensive sequential polarization design. Following *in vivo* implantation, the scaffold releases H_2_O_2_ over the initial 3 weeks to initiate chemodynamic therapy, which induces tumor cell apoptosis and ferroptosis. Concurrently, it activates an anti-tumor immune microenvironment by driving M1 macrophage polarization. Over the subsequent 12-week period, the sustained release of Mg^2+^ serves a dual function: it promotes the osteogenic differentiation of BMSCs by activating the Wnt3a/GSK-3β/β-catenin signaling pathway, and it fosters a favorable osteoinductive microenvironment by driving M2 macrophage polarization. This strategy deeply integrates anti-tumor therapy with immunomodulation, achieving a complete closed loop from active M1 induction to active M2 induction, thereby providing an ideal design paradigm for sequential polarization therapy of bone defects following tumor resection [154].

#### Age-associated bone defects

The fundamental challenge in age-associated bone defects lies in the systemic decline of the immuno-regenerative network rather than an imbalance in macrophage polarization per se. In the context of aging, dysregulated glycolytic and oxidative phosphorylation metabolism in macrophages induces aberrant succinate accumulation while concomitantly depleting intracellular NAD^+^ levels, ultimately leading to a loss of metabolic flexibility. This metabolic disturbance compromises the ability of macrophages to dynamically adapt their functional states in response to changing microenvironmental cues and markedly attenuates their sensitivity to polarization signals. Consequently, macrophage phagocytic activity is significantly impaired, resulting in reduced clearance efficiency of apoptotic cells and pathogens [155]. In addition, the persistent senescence-associated secretory phenotype (SASP), characterized by sustained secretion of pro-inflammatory cytokines such as IL-6 and TNF-α, establishes a state of chronic low-grade inflammation. This inflammatory milieu not only suppresses osteogenic differentiation but also further exacerbates macrophage dysfunction [156].

Unlike the ‘enhanced M1 response with impaired M2 transition’ observed under diabetic conditions, the ‘M1-locked’ state associated with infection, and the ‘M2-locked’ phenotype within the TME, the central issue under aging conditions is systemic deterioration. Insufficient M1 initiation leads to defective early-stage host defense, inefficient M2 responses result in delayed tissue repair, and SASP-driven chronic inflammation persists throughout the entire process. In view of these pathological characteristics, sequential polarization strategies for age-associated bone defect should be positioned as a ‘systemic restoration’ paradigm. This requires the use of persistently reinforced polarization cues, such as increasing the local concentration of inductive factors, prolonging their duration of action, and applying multi-target synergistic stimulation. Concurrently, senescent cells should be eliminated, and macrophage metabolic flexibility should be restored through metabolic reprogramming.

For the regulation of macrophage activity in age-associated bone defect, Alhamdi *et al*. used a bCaP coating to spatially segregate IFN-γ from the stimulatory agent simvastatin (SIMV). Following implantation *in vivo*, IFN-γ and SIMV were sequentially released from the bCaP coating, thereby enabling a macrophage polarization sequence from an initial M1 phenotype to a subsequent M2 phenotype. However, whether the M1 and M2 phenotypes promoted by the bCaP delivery system can induce skeletal repair in aged mice remains to be elucidated [97]. To address the impaired capacity of macrophages to transition from the M1 to the M2 phenotype in aged individuals, Fukuda *et al*. developed phosphatidylserine liposomes (PSLs). As an age- and sex-independent inducer of M2 macrophages, PSLs induced macrophage conversion toward the M2 phenotype with comparable efficiency in aged mice (>21 months old) and young mice [157]. Inspired by concrete structures, Li *et al*. developed a biomimetic bone glue that significantly enhances osseointegration and simultaneously promotes both early and late osteogenesis in osteoporotic bone defects. Transcriptomic analysis demonstrated that the bone glue could induce M2 polarization of senescent macrophages by inhibiting the NF-κB signaling pathway, thereby accelerating bone defect repair and regeneration [158]. Studies have shown that, during the repair of aging-associated bone injuries, the expression of triggering receptor expressed on myeloid cells 2 (TREM2) is downregulated, resulting in impaired inflammatory regulation [159]. Nevertheless, whether the above-mentioned strategies, which directly bypass the M1 phase and forcibly establish an M2-dominated reparative microenvironment, weaken early debridement capacity and interfere with initial endogenous recruitment signals remains to be further investigated.

Although bone defect repair under the above-mentioned pathological conditions is complicated by multiple overlapping factors, the sequential model in which M1 macrophages predominantly mediate inflammatory clearance and M2 macrophages primarily govern tissue repair remains applicable to the core process of bone regeneration. However, under distinct pathological backgrounds, the release profile and intensity of polarization cues within sequential polarization strategies should be adjusted and optimized to accommodate specific changes in the microenvironment. Notably, local implantation materials alone are often insufficient to reverse systemic metabolic or immune disorders. Therefore, the synergistic integration of systemic pharmacotherapy and local bone repair materials should be emphasized. For example, underlying pathological conditions may first be controlled using agents such as hypoglycemic drugs or antibiotics, followed by implantation of bone repair materials to achieve spatiotemporally precise immunomodulation and osteogenic induction.

### Clinical transition and regulatory considerations

Although the above-described regulatory strategies based on sequential macrophage polarization have demonstrated bone repair potential in various animal models, their successful translation into clinical products still depends on a systematic evaluation of complex factors, including biosafety, manufacturing processes and regulatory pathways. The limited physiological relevance of *in vitro* models, together with the substantial differences in bone healing kinetics between rodents and humans, represents a primary obstacle in translational research. In addition, the scalable production of materials that simultaneously maintain immunomodulatory functionality and batch-to-batch consistency remains a critical bottleneck that must be overcome during the transition from laboratory studies to clinical application. Therefore, a comprehensive analysis of these translational challenges is of great significance for guiding the design and evaluation of next-generation osteoimmunomodulatory materials.

Regarding material safety, although most base materials have been demonstrated to possess favorable biocompatibility, the bioactive components incorporated within them, such as cytokines and nanoparticles, still require rigorous immunological safety evaluation. Exogenous cytokines, including IFN-γ and IL-4, may elicit host immune responses, leading to attenuated therapeutic efficacy or even severe adverse reactions [160]. Nanoparticles at high concentrations or composed of specific materials may exert cytotoxic effects on host cells, while inappropriate particle size or surface properties may result in their accumulation in organs such as the liver and spleen, thereby causing organ damage [161]. In addition, the long-term degradation behavior of materials and the *in vivo* fate of their metabolic products, including released ions and nanoscale debris, should be systematically assessed to exclude potential risks of genotoxicity or long-term chronic inflammation. A moderate degradation rate enables composite materials to maintain structural integrity during degradation and fully exert their immunomodulatory functions. Conversely, materials with uncontrolled degradation kinetics may lead to the release of degradation-associated molecular patterns, activate the NLRP3 inflammasome, trigger foreign body reactions and compromise the spatiotemporal precision of sequential polarization.

Sequential polarization strategies carry an inherent risk of failure during *in vivo* applications. Most existing immunomodulatory materials rely on chemical cues, such as cytokines and bioactive ions, to achieve the temporal regulation of macrophage phenotypes from an initial M1 state to a subsequent M2 state. However, growth factors and cytokines loaded onto biomaterials suffer from short half-lives, poor stability and are prone to rapid clearance or inactivation *in vivo* [162], which may compromise or even negate the intended efficacy of implantable materials in practical applications. The degradation behavior of materials can also directly affect the immunomodulatory efficacy of implantable materials. If the material degrades too rapidly, the subsequent M2 induction phase may fail due to the lack of sustained signals; conversely, if degradation is too slow, residual material may trigger chronic inflammation or fibrous encapsulation [134]. Moreover, excessively rapid scaffold degradation may disrupt the macrophage polarization process, ultimately leading to the failure of bone healing *in vivo* [163]. Overall, the success of sequential polarization depends on precise alignment between the timing of M1-to-M2 transition and the natural progression of bone repair. This requires systematic coordination among the material degradation rate, drug release kinetics and the temporal window of M1/M2 phenotypic conversion during material design.

Batch-to-batch variability during scaled-up production represents one of the major obstacles to the clinical translation of bone repair materials. Natural materials, such as gelatin, collagen, alginate and other biomacromolecules, inherently exhibit batch-dependent variations. Their chemical composition, molecular weight distribution and gelation behavior may differ according to their source and extraction process. After chemical modification, such variability is often further amplified, resulting in inconsistencies among different batches in terms of mechanical properties, degradation kinetics and immunomodulatory efficacy [164]. Batch-to-batch variability in immunomodulatory bioactive components, such as cytokines and exosomes, is equally challenging. Paganini *et al*. pointed out that conventional methods for extracellular vesicles (EVs) preparation generally suffer from low yield and substantial batch-to-batch variability. Even under identical cell culture conditions, different culture modes and harvesting strategies can still affect the yield and quality attributes of EVs [165]. Therefore, recombinant or synthetic analogs may be required to replace naturally extracted components, thereby reducing raw material variability, while standardized synthetic processes should be developed to control the consistency of modification degrees. In addition, a systematic batch-to-batch functional validation framework should be established to ensure that product performance meets predefined quality specifications.

In addition, the regulatory pathway for the clinical translation of sequential polarization-based bone repair materials is highly complex. Such materials typically integrate multiple components, including scaffold carriers, bioactive ions and cytokines, and possess both medical device- and drug-like attributes; thus, they are generally classified as combination products. Their review and approval pathway is complicated, and no approved precedent has yet been established. Current registration pathways are determined according to the primary mode of action (PMOA). If the immunomodulatory function is primarily driven by released biologics, the product may be regulated through the biologics pathway; if the structural support function of the material predominates, it may instead be regulated as a medical device. Therefore, the determination of PMOA and the selection of the appropriate registration pathway should be clarified at an early stage of product development, and the safety profile of the material should be validated in early-phase clinical trials to accumulate evidence supporting regulatory decision-making [166].

Future clinical translation should integrate microphysiological systems, organ-on-a-chip platforms and artificial intelligence-assisted high-throughput screening platforms to bridge the gap between animal models and human physiology, thereby enabling precise translation from basic research to clinical application. Early-phase clinical trials should prioritize infection control, including clinical signs, microbial culture results and reoperation rates, as well as safety and functional/composite endpoints. Moreover, such studies should strictly employ materials manufactured under conditions representative of the final product.

## Challenges and future perspectives

Bone repair materials capable of sequentially modulating macrophage polarization represent a frontier direction in bone tissue engineering. By mimicking the dynamic variation in the immune microenvironment during the inflammatory and reparative stages of bone repair, these materials successively induce the pro-inflammatory M1 phenotype and the pro-reparative M2 phenotype, thereby achieving efficient coupling of angiogenesis and osteogenesis. Nevertheless, such materials still face numerous challenges in both fundamental research and clinical translation.

### Insufficient understanding of the timeline for bone immune repair

**Figure 7:**
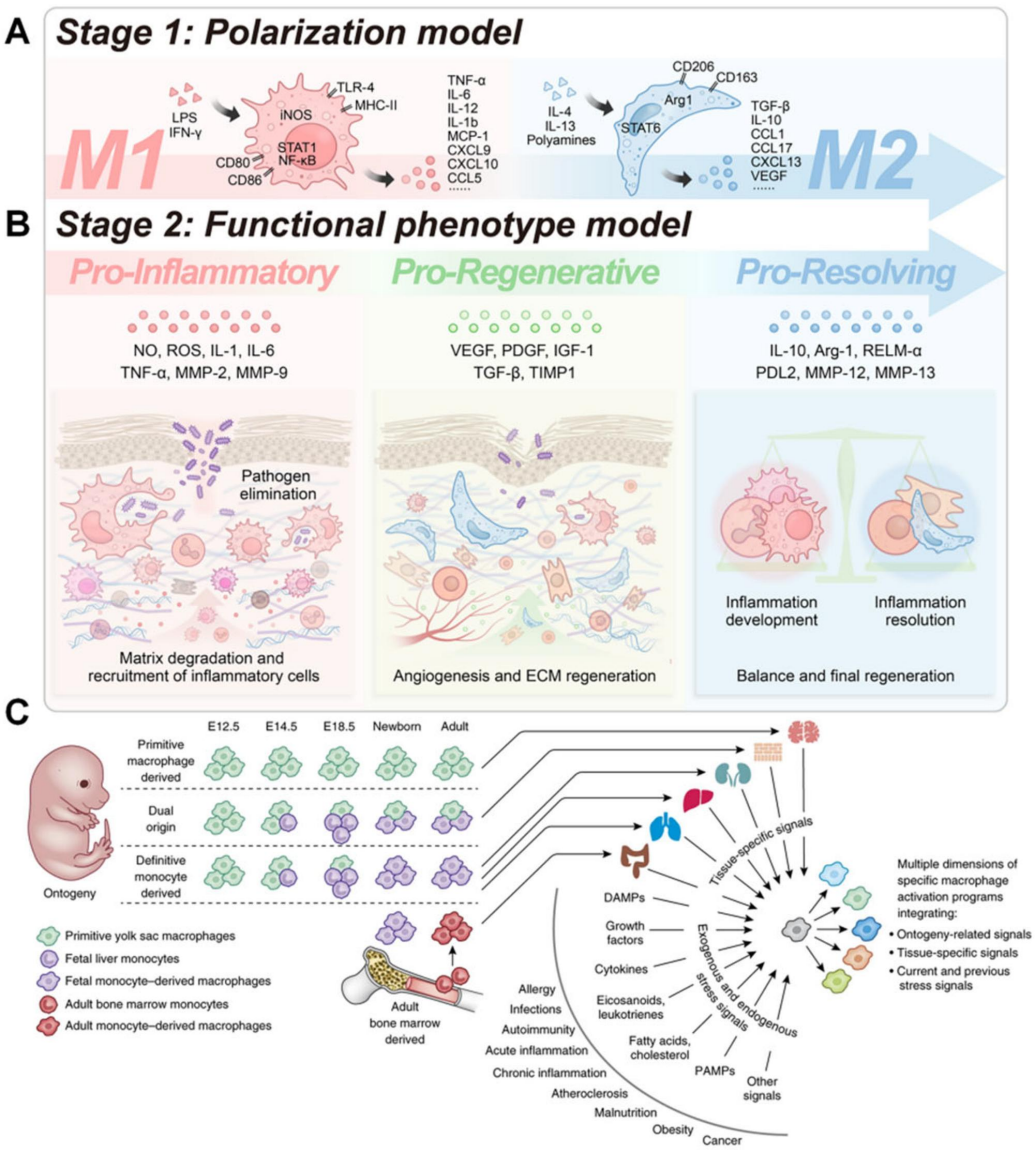
Evolution of macrophage polarization models: from binary classification to a multidimensional activation spectrum. (**A**) Simplified M1 and M2 polarization model. (**B**) Functional phenotype model based on macrophage transcriptomic and functional profiling. (**C**) A comprehensive multidimensional macrophage activation spectrum incorporating ontogeny, local tissue microenvironment and stress signals [167].

The classification of M1 and M2 macrophages has helped simplify our understanding of macrophage function in pathology and has guided the development of OIM materials for many years (Figure 7A). However, in reality, M1 and M2 macrophages are not simply binary opposites.

First, from a taxonomic perspective, macrophages are not limited to only M1 and M2 polarization states. Although the M2 macrophage lineage encompasses further subdivisions, such as M2a, M2b, M2c and M2d [168], this classification still falls short of capturing their full diversity. In fact, macrophages may exist in transitional states between the two polarization extremes; for instance, some cells exhibit characteristics of both M1 and M2 phenotypes or co-express markers of both types [169]. This hypothesis of a continuous polarization spectrum has received experimental support. Jia *et al*. [170] obtained transcriptomic datasets from 299 macrophages under different activation stimuli and thereby extended the M1/M2 polarization model into a continuous activation spectrum. Palma *et al*. [113] employed gene regulatory networks (GRNs) to model the continuity of macrophage polarization between the M1 and M2 poles. Similarly, Liu *et al*. constructed computational models to predict, beyond the classical M1-M2 polarization axis, a secondary axis of macrophage activation in response to combinatorial stimuli, simulating how macrophages respond to conflicting polarization cues [171]; such large-scale flexible functional responses represent a strategy employed by macrophages to navigate conflicting environmental cues [172].

Second, from a functional perspective, simply defining macrophages as either the M1 (pro-inflammatory) or M2 (anti-inflammatory) phenotype is reductive (Figure 7B). Even the different M2 subtypes, such as M2a, M2b, M2c, M2d and M2f, exhibit significant transcriptomic differences and perform diverse functions. For instance, during angiogenesis, PDGF-BB, which stabilizes pericytes and nascent blood vessels, is secreted by M2a macrophages, whereas M2c macrophages secrete substantial amounts of MMPs involved in vascular remodeling and also secrete osteopontin to immunomodulate EC proliferation, migration and vascular formation. Meanwhile, M2f macrophages markedly express TGF-β1, thereby enhancing vascular stability and promoting EC migration *in vitro* and vascular formation *in vivo* [17, 39]. Furthermore, although M2 macrophages are generally considered to contribute positively to bone regeneration, it remains unclear which specific subtypes are involved in this process, and the precise role of each subtype during bone regeneration has yet to be fully elucidated.

This functional subset differentiation beyond the conventional M1/M2 axis has been systematically validated across multiple fields. In liver metastases, an integrative multi-omics study identified the key metabolic molecule SLC2A1 as a driver of immunosuppressive microenvironment formation by increasing the proportion of immunosuppressive Spp1^+^ macrophages and enhancing their interactions with T cells [173]. In glioblastoma, Kloosterman *et al*. identified a functionally distinct subset of lipid-laden macrophages (LLMs). This subset neither strictly conforms to M1 characteristics, as reflected by low expression of inflammatory pathway genes, nor fully fits the M2 classification, as evidenced by high expression of lipid metabolism-associated genes such as Lp1 and Lipa. Instead, its functional specialization is characterized by phagocytosis of myelin debris and transfer of lipids to tumor cells via the LXR/Abca1 pathway to support their high metabolic demands [174]. In tendon injury and regeneration, Zhang *et al*. analyzed the transcriptomes of more than 74 000 human single cells and identified FOLR^2+^ macrophages with anti-fibrotic functions. This population exhibits a distinct phenotype and well-defined function that cannot be adequately captured within the M1/M2 framework [175].

In summary, during physiological tissue repair, macrophages commonly adopt intermediate or mixed polarization states, and their dynamic polarization is continuously orchestrated by multiple interdependent factors, such as cytokines, extracellular matrix stiffness, and cellular metabolic status. Targeting only the M1 and M2 phenotypes fails to capture the true dynamic changes of macrophages [176]. Therefore, a simplistic M1/M2 classification should be abandoned in favor of an integrated approach that considers the tissue origin and specific environmental stimuli of macrophages, combining their functional and transcriptomic profiles to establish a unified functional network model of macrophages [177] (Figure 7C). This perspective, which extends beyond the binary M1/M2 classification, has been validated in tumor immunology. A single-cell transcriptomic study of uveal melanoma identified four macrophage subsets, among which MΦ-C4 exhibited low expression of both M1- and M2-associated signature genes and showed loss of inflammatory pathway activity and antigen-presenting functions, but was instead enriched in signals related to proliferation, mitochondrial function and metabolism [178]. Similarly, in the field of bone repair, future studies should employ multi-omics integration approaches, including single-cell RNA sequencing, spatial transcriptomics, proteomics and metabolomics, together with isotope tracing techniques, to delineate the dynamic changes in bona fide functional macrophage subsets at different stages of bone healing and construct a comprehensive atlas of macrophage metabolism. Such efforts will provide guidance for the design of more precise temporally programmed immunomodulatory materials.

### Precision assessment of *in vivo* macrophage polarization

Unlike *in vitro* culture environments, where LPS combined with IFN-γ or IL-4 serves as a potent stimulus to drive macrophage commitment toward M1 and M2 phenotypes, respectively, macrophage phenotypes under *in vivo* physiological conditions exhibit significant heterogeneity, with such differences observed across multiple tissues, including bone, blood vessels, heart and brain [179]. As emphasized by Camille *et al*. [180], the characteristics and functions of tissue-resident macrophages depend on four factors: ontogeny, local environment, inflammatory status and time, enabling them to respond to ever-changing external cues. In the acute peritonitis microenvironment, Bystrom *et al*. [181] isolated macrophages from a mouse model during the resolving stage of inflammation. These macrophages expressed M2 markers such as the mannose receptor, IL-10, and Arg-1, while still maintaining low expression of M1 markers including cyclooxygenase-2 (COX-2) and iNOS, a phenomenon that deviates from the M1/M2 classification established by *in vitro* experiments. Furthermore, in the pathological microenvironment of osteoarthritis, synovial macrophages exhibit concurrently enhanced phagocytic and immunosuppressive functions, and their surface marker expression does not fully conform to the classical M1/M2 phenotypes defined *in vitro* [182]. Regarding the reason why most surface markers used to identify macrophages *in vitro* are not applicable *in vivo*, Orecchioni *et al*. [183] systematically compared the *in vivo* transcriptome of mouse macrophages with the *in vitro* transcriptome of BMDMs polarized by LPS+IFN-γ or IL-4 from other studies. The results showed that while *in vitro* and *in vivo* macrophages share certain polarization pathways and exhibit similar gene expression trends, there also exist many unique *in vitro* or *in vivo* pathways, in which many genes are either oppositely regulated or show no correlation. Therefore, it is essential to discover new, unified, and effective surface markers for the classification of macrophages in both *in vitro* and *in vivo* settings.

Beyond the discrepancies between *in vitro* and *in vivo* phenotypes, the phenotype, function and transcriptome of macrophages also undergo continuous changes over time. Although Mouton *et al*. [184] reported the macrophage landscape on days 1, 3 and 7 after myocardial infarction, the temporal dynamics of macrophages during bone repair in the microenvironment remain poorly understood. Notably, current related studies remain largely confined to *in vitro* experimental settings, and an in-depth understanding of the continuous transcriptomic changes during this process is still lacking. Mainstream studies indicate that M1 macrophages primarily function in the initial phase of bone healing (i.e. within 1–3 days), whereas M2 macrophages become active after 3 days. Strong but transient pro-inflammatory signals from early M1 macrophages effectively promote osteogenesis. A study by Loi *et al*. [133] demonstrated that, compared with intervals of 24 and 48 h, co-culturing M1 macrophages with MC3T3 cells for 72 h followed by an IL-4-induced transition from the M1 to M2 phenotype resulted in the release of greater amounts of OSM, a signaling molecule that promotes bone formation, and significantly enhanced osteogenic outcomes. Nathan *et al*. [16] also confirmed that a pro-inflammatory environment lasting 72–96 h is critical for optimal bone matrix mineralization. However, some studies have reported conflicting conclusions. Spiller *et al*. [17], while investigating the effect of macrophages on angiogenesis, found that a 1-day persistence of the M1 macrophage phenotype increased the formation of vascular structures following tissue-engineered vascular network implantation, whereas a 3-day persistence led to vascular regression. Moreover, although *in vitro* experiments have validated the notion that the same population of macrophages can transition between M1 and M2 phenotypes under specific stimuli, the precise origin of M2 macrophages in the *in vivo* bone repair microenvironment remains unclear. It is still unknown whether M2 macrophages arise from the repolarization of M1 macrophages, derive from the polarization of M0 macrophages upon migration to the injury site, or both scenarios are possible [14].

In summary, conclusions drawn from *in vitro* studies of macrophages cannot be directly applied to the phenotypes and functions of macrophages in the *in vivo* physiological microenvironment. *In vivo*, macrophages are subjected to combinatorial stimulation by tissue-specific signals and complex temporal cues; their phenotypes and functions are dictated by the specific tissue location, distinct microenvironment, and local stimuli [185], and may even respond to tissue developmental processes, ultimately coordinating their functions with those of the tissue. Therefore, precise assessment is essential to characterize the phenotype, function and transcriptome of macrophages in the *in vivo* bone defect microenvironment, in order to elucidate the specific roles and contributions of macrophages along the timeline of bone repair.

### Design principles for temporally programmed materials

Based on the aforementioned comparative summary of various sequential polarization strategies and the systematic analysis of mechanisms underlying macrophage polarization imbalance under different pathological conditions, ideal osteoimmunomodulatory repair materials intended for clinical application should follow the following design principles: first, the delivery of polarization cues should be coordinated with the natural progression of bone repair; second, precise regulation should be achieved through the synergistic integration of physical and chemical cues; and third, material design should be tailored to the specific characteristics of distinct pathological microenvironments.

First, regardless of the pathological context, the fundamental logic of sequential macrophage polarization remains the induction of an initial M1 phenotype followed by transition toward an M2 phenotype. Material design should ensure that, during the early stage after implantation (0–3 days), the local microenvironment is dominated by M1 macrophages to initiate the necessary inflammatory response and tissue debridement. Subsequently, during the reparative phase (3–14 days), M2 macrophages should exert sustained effects to promote inflammation resolution and osteogenesis. The release profiles of these two types of signals should avoid substantial overlap or temporal inversion. Based on the 3–7 day transition window, the physicochemical properties of materials, such as composition, crosslinking density, porosity and pore size, can be tailored to match the degradation kinetics accordingly. Rapidly degradable components may correspond to the M1-dominated phase and release pro-inflammatory regulatory factors; moderately degradable components may match the phenotypic transition window and release M2 polarization-inducing factors; and slowly degradable components may serve as sustained-release reservoirs for osteogenic factors, while smart responsive systems can be incorporated to enable on-demand release of regulatory cues. Premature release of M2-polarizing signals should be prevented to avoid compromising the debridement or antibacterial efficacy of the material, while prolonged release of M1-polarizing signals should also be avoided to prevent inhibition of tissue repair. Precise temporal matching is the primary criterion for evaluating the rationality of material design. Ideally, such materials should mimic the immune timeline of natural bone repair and achieve a smooth transition between the two macrophage phenotypes.

Second, for osteoimmunomodulatory repair materials, the intrinsic physicochemical properties of the material itself, including stiffness, surface topography, porosity, hydrophilicity/hydrophobicity and degradation behavior, also play indispensable roles in regulating macrophage polarization. Therefore, material design should systematically integrate these physical cues, comprehensively considering their inherent capacity to regulate immune cell fate as well as their synergistic or antagonistic interactions with chemical cues, such as cytokines, drugs and bioactive ions. Notably, the effects of physical and chemical cues on macrophage polarization are not independent of each other but instead involve complex interactions. An in-depth elucidation of the structure–function relationships between these two types of cues is a critical prerequisite for optimizing the immunomodulatory performance of materials. On this basis, researchers should fully exploit the fundamental and sustained immunoregulatory signals provided by physical cues, while leveraging the programmable and temporally reinforced signals afforded by chemical cues. Through the organic integration and synergistic optimization of these two regulatory systems, precise and efficient control over macrophage polarization behavior can be achieved, ultimately leading to optimal osteoimmunomodulatory repair outcomes.

Finally, fixed temporal strategies that are validated as effective in healthy animal models often show markedly reduced efficacy under pathological conditions. Therefore, for bone defect repair under different pathological backgrounds, the sequential polarization strategy of implanted materials should be promptly adjusted according to the central pathophysiological challenge of the specific disease etiology. For diabetic bone defects, glucose-lowering and antioxidant modules should be integrated, while the intensity and duration of M2-inducing signals should be substantially enhanced. For infected bone defects, pathogen clearance should be prioritized, and an on-demand switching mechanism should be adopted, whereby the initiation of M2 signals is delayed until infection is adequately controlled to avoid compromising antibacterial efficacy. For post-tumor resection bone defects, priority should be given to eliminating residual tumor cells and reprogramming TAMs to induce their conversion from the M2 phenotype to the M1 phenotype, thereby establishing a local microenvironment dominated by anti-tumor immunity. For age-related bone defects, senescent cell clearance and metabolic reprogramming modules should be incorporated, and persistently reinforced polarization cues should be used to restore the functional responsiveness of macrophages. In addition, under pathological conditions, the M1/M2 transition window exhibits pronounced interindividual variability and temporal uncertainty. Material design should therefore introduce dynamic responsive mechanisms that use microenvironmental signatures, such as pH, ROS levels and enzymatic activity, as endogenous triggers, or integrate external field-based regulatory approaches, such as magnetic fields, near-infrared light and ultrasound, to achieve on-demand release of polarization cues.

### Future development direction

Based on existing challenges and design principles oriented toward clinical translation, future osteoimmune materials with programmable macrophage-modulating functions should adopt smart design concepts closely aligned with clinical practice, advancing towards a developmental paradigm that deeply integrates environmental responsiveness, data-driven approaches, and artificial intelligence-assisted decision-making. Specifically, smart material systems highly sensitive to pathological microenvironmental signals should be introduced to achieve the spatiotemporally controlled release of polarizing factors. Simultaneously, integrating high-throughput experimental platforms with AI algorithms will enable the unearthing of underlying correlations between material properties and immune responses, thereby facilitating the construction of material-immune response predictive models. Ultimately, these efforts will culminate in the development of next-generation osteoimmune-modulating materials that boast precision, adaptability and immense potential for clinical translation.

Future osteoimmune-modulating materials should break through the traditional framework of preset sequential delivery, advancing toward a new developmental paradigm of adaptive materials. This necessitates the construction of intelligent decision-making systems driven by the pathological microenvironment, which can be achieved by introducing bioorthogonal chemically responsive units and signal amplification mechanisms to form a closed-loop feedback regulatory circuit. Implanted materials must possess the capability to perceive dynamic changes in local biomarker concentrations in real time and trigger the on-demand release of factors through negative feedback mechanisms, actively reconstructing the immune homeostasis of the bone microenvironment, thereby facilitating a paradigm shift from passive delivery to active modulation [186]. Simultaneously, in synergy with external field intervention strategies such as magnetic fields and near-infrared irradiation, comprehensive and precise control over the chronological progression of macrophage polarization can be further realized.

Undeniably, the ultimate objective of material development is clinical application, which necessitates the establishment of a multi-tiered, highly biomimetic preclinical validation system. Existing models are inadequate in comprehensively reflecting complex pathological conditions involving metabolic dysregulation, such as aging and diabetes. Even when animal models closely matching the pathological background of the target indications are employed, fully recapitulating the intricate biological dynamic processes and individual variations inherent in the human body remains a formidable challenge [18]. To bridge the assessment bias arising from interspecies differences, advanced *in vitro* models, such as organ-on-a-chip and bone organoids, should be introduced. Organ-on-a-chip technology enables the simulation of key characteristics of the human osteoimmune microenvironment on microfluidic platforms, whereas bone organoids achieve the biomimetic reconstruction of the microenvironment through cellular self-assembly. These two models complement each other, providing an experimental vehicle that more closely mimics human physiology for the developmental validation and safety assessment of novel osteoimmune-modulating materials.

The rapid development of artificial intelligence (AI) is also propelling the transition of osteoimmune-modulating materials from an experience-driven to a data-driven paradigm. By integrating multidisciplinary knowledge across chemistry, physics and engineering, AI can unearth unconventional material combinations that are beyond the reach of traditional theoretical design, and inversely deduce and generate material structures based on preset performance targets [186]. Based on key parameters such as material composition, cross-linking density and degradation characteristics, AI models can achieve precise predictions regarding the sequential release behaviors of cytokines or bioactive ions, thereby significantly reducing trial-and-error costs in experiments. Furthermore, AI models trained on patients’ clinical data can recommend personalized sequential polarization regimens according to indices such as the duration of the patient’s diabetes and the severity of infection, facilitating precision immunomodulation.

The efficient research and development of future osteoimmune-modulating materials rely on the construction of high-throughput screening platforms that integrate microfluidic chips, automated synthesis and multi-parameter characterization technologies. Such platforms can systematically evaluate the synergistic effects of multidimensional parameters, which include material components, topological structures, and mechanical properties, on macrophage polarization within a single experimental framework. Specifically, microfluidic technology enables cell culture and real-time monitoring with minimal sample consumption; automated modules support the rapid preparation of material arrays; and multi-parameter characterization systems synchronously acquire key indices such as cellular phenotypes, inflammatory factors and material degradation. Based on the high-quality data accumulated from the aforementioned experimental systems, a material property-immune response database can be further constructed. Aided by AI algorithms, this approach can efficiently identify key combinations of material characteristics that drive M2 polarization, realizing the rapid screening and iterative optimization of candidate materials. The deep integration of high-throughput automated experimental platforms and AI holds immense promise to significantly accelerate the research and development process of osteoimmune-modulating materials and reduce trial-and-error costs.

In summary, the core of future development for sequentially polarizing osteorepair materials lies in constructing an intelligent decision-making system driven by the pathological microenvironment. This approach aims to achieve the precise chronological reprogramming of macrophage polarization through closed-loop feedback regulation, facilitating a paradigm shift from preset delivery to adaptive modulation. Furthermore, the synergistic application of AI-assisted material design, high-throughput screening platforms, and organ-on-a-chip validation models provides a potential pathway to resolve the mismatch dilemma between the intelligent attributes of materials and current regulatory frameworks. Ultimately, the synergistic advancement of various strategies is essential to enable the sequential modulation strategy to achieve the leap from the laboratory to clinical application, truly serving the clinical repair of complex bone defects.

## Conclusion

Macrophages are indispensable for maintaining bone homeostasis and orchestrating the complex cellular cascade that involves MSCs, ECs, and osteoblasts throughout the entire bone repair continuum. The spatiotemporal dynamic of their phenotypic polarization, particularly the sequential transition from early pro-inflammatory M1 phenotypes to subsequent pro-reparative M2 phenotypes, dictates the trajectory of bone healing. Specifically, a robust initial M1 phase is critical for pathogen clearance and tissue debridement, whereas a timely transition to the M2 phenotype is prerequisite for initiating osteogenesis and angiogenesis. Conversely, dysregulation of this critical M1-to-M2 switch frequently culminates in chronic inflammation, pathological fibrosis or even atrophic nonunion.

Guided by these biological paradigms, next-generation OIM biomaterials are being rationally designed to emulate the natural healing cascade. By leveraging precision-engineered release kinetics, hierarchical spatial architectures, and physicochemical stimulus-responsiveness, these constructs enable the spatiotemporally controlled sequential polarization of macrophages, thereby profoundly enhancing angiogenic-osteogenic coupling.

Despite these promising advances, several formidable challenges impede rapid clinical translation. The inherent functional diversity, extreme heterogeneity and plasticity of macrophages complicate the full elucidation of their roles. Furthermore, profound discrepancies in phenotypic markers and transcriptomic profiles persist between *in vitro* models and complex *in vivo* environments. From a materials perspective, precisely synchronizing the degradation kinetics and polarization signal release profiles of OIM biomaterials with the dynamic *in vivo* immune timeline remains a significant technical hurdle.

Looking forward, the integration of high-resolution single-cell multi-omics, spatial transcriptomics and mass cytometry will unequivocally map the origin, activation trajectories and precise functions of macrophages within the bone defect microenvironment. The establishment of comprehensive *in vivo* macrophage functional atlases will fundamentally enhance our capacity for precise immunomodulation. Ultimately, this will catalyze the development of sophisticated, macrophage-targeted OIM biomaterials, accelerating their clinical translation to robust skeletal regeneration.
